# Comparative analysis of sperm preparation techniques on DNA fragmentation and clinical outcomes: a network meta-analysis

**DOI:** 10.3389/fendo.2026.1817587

**Published:** 2026-07-13

**Authors:** Linlin Wang, Jingjing Xu, Yaman Guo, Xintao Wang, Meng Jia

**Affiliations:** 1 Independent researcher, Leiden, Netherlands; 2 The Longhu Community Medical Service Centre, Xinzheng, Henan, China; 3 Henan Province People’s Hospital, Zhengzhou, Henan, China; 4 The First Affiliated Hospital of Zhengzhou University, Zhengzhou, Henan, China

**Keywords:** clinical outcomes, magnetic-activated cell sorting, microfluidic sperm sorting, sperm DNA fragmentation, sperm preparation technique

## Abstract

**Introduction:**

Elevated sperm DNA fragmentation negatively affects assisted reproductive technology outcomes. Although multiple sperm preparation techniques are used, existing evidence is largely limited to pairwise comparisons. We performed a systematic review and network meta-analysis to investigate the most effective technique for reducing sperm DNA fragmentation and improving clinical outcomes.

**Methods:**

PubMed, Embase and Cochrane Central Register of Controlled Trials were searched to July 26, 2025. Due to the limited randomized studies for sperm DNA fragmentation (N=2), both randomized and non-randomized studies comparing at least two preparation techniques were included for sperm DNA fragmentation analyses, while only randomized controlled trials were included for clinical outcomes to ensure the highest stringency for treatment efficacy. Data were synthesized using frequentist random-effect network meta-analysis.

**Results:**

Forty-two studies (2697 semen samples) were included for sperm DNA fragmentation. Despite high heterogeneity (*I^2^*=*96.1%*), network estimates indicated that microfluidic sperm sorting (MFSS) was associated with a significant showed the greatest reduction in DNA fragmentation (SMD -2.64; 95%CI -3.53 to -1.75), followed by magnetic-activated cell sorting (MACS) combined with density gradient centrifugation (DGC; SMD, -1.53; 95%CI -2.93 to -0.12). Fifteen randomized controlled trials reported clinical outcomes. Magnetic-activated cell sorting combined with density gradient centrifugation was associated with a higher clinical pregnancy rate compared with density gradient centrifugation alone (OR1.93; 95% CI 1.23 --3.02), and pairwise meta-analysis showed a lower miscarriage rate (OR 0.65; 95% CI 0.46 -0.91).

**Discussion:**

While MFSS and MACS-based combinations showed the greatest numerical reduction in sperm DNA fragmentation, these findings are characterized by high heterogeneity and low certainty of evidence. Although some improvements in clinical pregnancy and miscarriage rates were observed with the combination of MACS and DGC, the lack of robust, large-scale data prevents a definitive clinical recommendation outcomes.

**Systematic Review Registration:**

https://www.crd.york.ac.uk/PROSPERO/view/CRD420251181955, identifier CRD420251181955.

## 1 Introduction

Sperm DNA integrity is increasingly recognized as a critical determinant of male fertility, and may provide additional diagnostic and prognostic information beyond conventional semen parameters (1). Elevated sperm DNA fragmentation (SDF) has been associated with impaired embryo development, increased miscarriage risk, and reduced pregnancy rates in both natural conception and assisted reproductive technology (ART) cycles (2, 3). Accordingly, both American Urological Association (AUA) (4) and the European Association of Urology (EAU) (5) have highlighted the clinical relevance of SDF assessment as a biomarker in selected infertile populations.

Swim-up (SU) and density gradient centrifugation (DGC) remain the most widely used sperm preparation techniques in ART laboratories. However, both techniques have limited ability to effectively removing DNA-damaged spermatozoa (2) (6). To improve sperm selection, Magnetic-activated cell sorting (MACS) was introduced to remove apoptotic spermatozoa via annexin V binding. By delimiting apoptotic sperm, MACS reduce the proportion of DNA-damaged sperm in the selected fraction and has been associated with improved embryo quality (7, 8), higher clinical pregnancy rates, and lower miscarriage rates (8). Nevertheless, other studies have reported modest or non-significant reductions in SDF following MACS (9), indicating that DNA-damaged spermatozoa may still persist in the MACS-selected fraction.

Combining MACS with conventional sperm preparation techniques has therefore been proposed, with studies reporting higher motility, improved viability and reduced apoptotic markers compared with MACS alone (10, 11). However, the optimal sequencing of DGC and MACS remains controversial. Tavalaee et al. (12) suggested that performing MACS before DGC may be more beneficial for clinical selection, whereas Chi et al. (13) argued that the limited loading capacity and small column size of MACS may compromise its efficiency when applied before DGC.

More recently, microfluidic sperm sorting (MFSS) has emerged as a centrifugation-free alternative that mimics aspects of the *in vivo* reproductive tract (14–16). Several studies have supported the effectiveness of MFSS in reducing SDF (16–20) and improving clinical outcomes. However, other studies found that MFSS did not provide much benefit over conventional sperm preparation techniques (20, 21).

Although multiple sperm preparation techniques are routinely used in ART to reduce SDF and potentially improve clinical outcomes. The relationship between SDF and reproductive outcomes remains inconclusive. In addition, given the increasing number of available sperm preparation techniques and limited comparisons among them, conventional pairwise meta-analysis may be insufficient to provide a comprehensive evaluation. A network meta-analysis is therefore essential to simultaneous comparison of multiple interventions by integrating both direct and indirect evidence. However, we release that the diversity of laboratory protocols, patient populations, and SDF assessment methods introduce potential heterogeneity; thus, our analysis employs rigorous methodology to account for these variables.

This network meta-analysis aims to compare DNA fragmentation index (DFI) as a primary outcome and clinical outcomes as secondary outcomes across SU, DGC, MACS, their combinations and MFSS.

## 2 Methods

### 2.1 Protocol and reporting

The protocol for this systematic review and network meta-analysis was registered on PROSPERO(CRD420251181955) https://www.crd.york.ac.uk/PROSPERO/view/CRD420251181955. The study was performed following the guidelines of the Preferred Reporting Items for Systematic Reviews and Meta-Analyses (PRISMA).

### 2.2 Search strategy

Two trained reviewers (Y-G and J-X) independently conducted a comprehensive literature search in PubMed, Embase and Cochrane Central Register of Controlled Trials (CENTRAL) to identify studies comparing sperm DNA fragmentation and clinical outcomes across the following sperm preparation techniques: SU, DGC, MACS, their combinations and MFSS. The search covered all records from database from inception to July 2025. Medical subject headings and keywords used in the search strategy are presented in **Supplementary Table 1**. Full text was independently reviewed by both reviewers. Differences were discussed until consensus could be reached. Reference lists of relevant reviews and meta-analyses were also manually screened to identify additional eligible studies.

### 2.3 Eligibility criteria

#### 2.3.1 Types of studies and participants

For the DFI analysis, both randomized and non-randomized studies were included, whereas only randomized controlled trials (RCTs) were included for clinical outcomes analyses. Only original full-text articles published in English were considered. Studies were included if they involved human semen samples from infertile or subfertile men, normozoospermic men undergoing fertility evaluation, or clinical sperm donors. Studies using semen samples from healthy volunteers recruited solely for research purpose were exclude.

#### 2.3.2 Types of interventions

Intervention classification was adapted according to outcome type. For DFI outcomes, where sufficient data were available, combined techniques were categorized based on the sequence of procedures. Specifically, MACS-DGC refers to MACS performed prior to DGC whereas DGC-MACS refers to DGC performed prior to MACS. Similarly, PSU-MACS indicates pellet swim-up performed before MACS and MACS-DSU indicates direct swim-up performed after MACS.

For clinical outcomes, due to limited study numbers and to preserve network connectivity, such sequence-based distinctions were not maintained. Instead, combined interventions were grouped into single cetegories.

DGC and SU were required to be performed according to the WHO guidelines (22). Swim-up techniques were further categorized as pellet swim-up (PSU) or direct swim-up (DSU) based on whether centrifugation preceded sperm migration. When swim-up was performed following DGC, the technique was classified as DGC-PSU.

Only commercially available laminar-flow microfluidic sperm-sorting systems (e.g., ZyMot, Qualis, LensHooke Ca0) were classified as MFSS.

Overall, studies were included if they compared DFI and/or clinical outcomes between at least two of the following techniques: MFSS, PSU, DSU, DGC, DGC-PSU, and MACS. For DFI outcomes, sequential combinations of MACS with DGC or SU were additionally considered and categorized based on procedural order as described above, whereas for clinical outcomes, such sequential distinctions were not applied.

### 2.4 Outcome measures

The primary outcome was DFI, defined as the percentage of spermatozoa with DNA fragment. DFI was assessed using validated assays, including TUNEL, sperm chromatin dispersion (SCD), sperm chromatin structure assay (SCSA), comet assay and toluidine blue staining. Studies were required to report outcomes as means and standard deviations (mean ± SD), or provide data convertible to this format.

Secondary outcomes included clinical pregnancy rate, live birth rate, miscarriage rate, implantation and fertilization rate. Clinical pregnancy rate is defined as the presence of fetal heartbeats at 6 to7 weeks of gestation. Miscarriage was defined as pregnancy loss before 20 weeks of gestation among clinically confirmed pregnancies (23).

### 2.5 Study appraisal and synthesis methods

Data extraction was performed independently by two reviewers (J-X and Y-G). Discrepancies were identified and resolved through a consensus-based discussion. Inter-rater agreement was high, with discrepancies occurring in only three studies, all of which were resolved by discussion with a third researcher (M-J). Extracted data for DFI outcomes included study characteristics, participant characteristics, sperm preparation techniques, DFI assay methods, sample size and reported outcomes. For clinical outcomes, extracted data included study design, sperm preparation techniques and ART treatment type.

When numerical data was not directly reported, values were extracted from the figures or contacted the corresponding authors. When mean and SD were unavailable, they were estimated using established methods described by Wan et al., 2014 (24).

### 2.6 Risk of bias assessment

Risk of bias for individual studies was assessed independently by two researchers (L-W and X-W). Non-randomized studies were evaluated using the Newcastle-Ottawa Scale (NOS) (25), and RCTs were assessed using the Cochrane Risk of Bias 2.0 (RoB 2) tool (26). Disagreements were resolved by discussion with a third researcher (M.-J). For non-randomized studies involved in DFI analysis, given that the DFI index is an immediate laboratory outcome, follow-up related items in the NOS were interpreted accordingly. Scores ≥8 were considered low risk of bias, 7moderate, and ≤6 high risk.

### 2.7 Data analysis

We conducted a frequentist network meta-analysis using *netmeta* package (27). For DFI outcomes, given the use of different assays to measure DFI, pooled effects were expressed as standardized mean difference (SMDs) and 95% confidence intervals (CIs) to allow comparability across studies. For clinical outcomes, odds ratios (ORs) with 95% CIs were calculated.

Network meta-analysis was conducted for outcomes supported by a connected evidence network, which included DFI and clinical pregnancy outcomes. For DFI, pairwise meta-analyses were additionally conducted to enable direct comparison. For live birth and miscarriage outcomes, pairwise meta-analyses were performed when sufficient data were available; otherwise, results were summarized descriptively.

Between-study heterogeneity was assessed using τ^2^ and the Higgins I^2^ statistic (28). Given substantial heterogeneity (*I^2^* > 70%), random-effects models were applied.

Subgroup analyses were conducted only for DFI outcomes, stratified by DFI assay type and semen quality (normozoospermia vs. non-normozoospermia). Influence-based sensitivity analysis was performed by sequentially excluding studies contributing most to heterogeneity and evaluating changes in between-study variance. Design-based sensitivity analysis was additionally conducted by excluding RCTs to evaluate the impact of study design on the stability of the network for DFI outcomes. Meta-regression analysis was performed to explore the study-level covariates, including baseline DFI, male age, raw semen parameters.

For the network meta-analysis of DFI, surface under the cumulative ranking curve (SUCRA) score was used to estimate the relative ranking of each sperm preparation technique (29), and P-scores was computed under random effect models (27). SUCRA was not calculated for the network meta-analysis of clinical pregnancy outcomes due to limited number of included studies (n=10).

Potential publication bias was assessed using comparation-adjusted funnel plots and Egger’s regression test. Transitivity was evaluated by examining the distributions of the key study characteristics, including sperm quality and assay type used for DFI measurement, across comparisons. Global heterogeneity and inconsistency were assessed using the design-by-treatment interaction model (30). Node-splitting method (31)was used to verify the inconsistencies between direct and indirect evidence.

The certainty of evidence for network estimate on SDF was evaluated using the Grading of Recommendations Assessment, Development and Evaluation framework (GRADE) (32)and implemented through the Confidence in Network Meta-analysis (CINeMA) platform (33).

## 3 Results

### 3.1 Characteristics of included studies for DNA fragmentation

The electronic search identified 365 records: 239 from PubMed. 1 from CENTRAL and 125 from EMBASE. After removal duplicates and screening, 102 full texts were assessed for eligibility, and 42 studies (2 RCTs and 40 non-randomized studies) were included.

**Figure 1:**
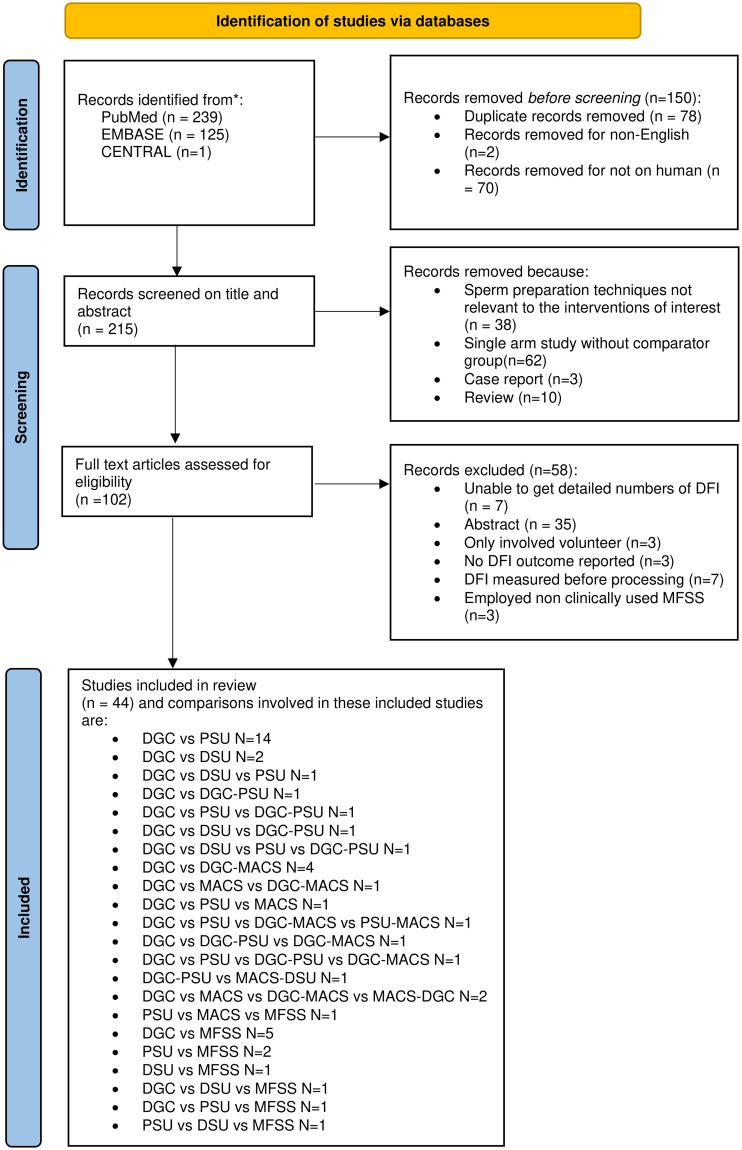
PRISMA flow diagram of article screening for sperm DNA fragmentation review. DGC, density gradient centrifugation; PSU, Pellet Swim-Up; DSU, Direct Swim-Up; DGC-PSU, Swim-Up after DGC; MACS, magnetic-activated cell sorting; MACS-DGC, DGC-MACS, PSU-MACS, MACS-WSU, sequential methods; MFSS, Microfluidic sperm sorting.

One small muti arm study (Wen et al., (24) was excluded from the network meta-analysis of DFI outcomes due to instability in variance estimation when applying calculating standardized mean differences. Overall, 42 studies comprising 2697 semen samples were included (**Table 1**; **Figure 1**).

**Table 1:** General characteristics of the studies included in the network meta-analysis for sperm DNA fragmentation.

First author (year)	Country	Study design	Methods compared	Sample size(N)	DFI assay	Key outcomes reported	Participant characteristics
Hsu et al., (17)	China	Prospective non-randomized	MFSS (Zymot) and DGC	119	SCD	DFI, post-selection concentration and post-selection progressively motility	Normozoopsermic (N = 35) Non-normozoospermic (N = 85)
Kishi et al., (18)	Japan	Prospective non-randomized	MFSS (Qualis) And PSU	10	SCD	DFI	^†^Mixed (Normal , oligo and asthenozoospemia)
Sheibak et al., 2024	Iran	Prospective non-randomized	PSU and DGC	100	SCD	DFI, post-selection concentration and Post-selection progressively motility	Normozoospermic(N = 50) Non-normozoospermic (N = 50)
Mirsanei et al., (34)	Iran	Prospective non-randomized	MFSS (Fertile Plus) and DGC	25	SCD	DFI, post-selection concentration and Post-selection progressively motility	Normozoospermic
Ješeta et al., (20)	Czech Republic	Prospective non-randomized	MFSS (Ca0 device by LensHooke/Bonraybio) and DSU	68	SCD	DFI, post-selection concentration and Post-selection progressively motility	Normozoospermic (N = 40) Non-normozoospermic(N = 28)
Parrella et al., 2019	USA	Prospective non-randomized	DGC and MFSS (Zymot)	48	TUNEL	DFI, post-selection concentration and Post-selection progressively motility	^†^Mixed
Traini et al., (14)	Italy	Prospective non-randomized	MFSS (Zymot), PSU and DSU	14	TUNEL	DFI and post-selection progressively motility	Normozoospermic
Yildiz et al., (35)	Turky	Prospective non-randomized	MFSS (Fertile Plus) and DGC	20 (N = 10 for DGC and N = 10 for MFSS)	Toluidine blue staining	DFI	^†^Mixed
Vahidi et al., (15)	Iran	Prospective non-randomized	MACS, MFSS(Zymot) and PSU	25	SCD	DFI and post-selection progressively motility	Non-normozoospemic
Zini et al., (6)	Canada	Prospective non-randomized	DSU and DGC	22	SCSA	DFI	^†^Mixed (Normospermic and oligospermic)
Xue et al., 2014	China	Prospective non-randomized	PSU and DGC	118	SCD	DFI	Teratozoospermic
M. Muratori et al., 2019	Italy	Prospective non-randomized	PSU and DGC	20with DGC and 40 with PSU	TUNEL	DFI and post-selection progressively motility	^†^Mixed
Amano et al., 2024	Japan	Prospective non-randomized	PSU and DGC	19	TUNEL	DFI and post-selection progressively motility	Normozoospermic
Kim et al., 2017	Korea	Prospective non-randomized	PSU and DGC	18	SCD	DFI	^†^Mixed
Amiri et al., 2012	Iran	Prospective non-randomized	PSU and DGC	35	Comet assay	DFI and post-selection concentration	^†^Mixed
Viswambharan et al., 2020	India	Prospective non-randomized	PSU and DGC	32	SCD	DFI	Normozoospermic
Jayaramen et al., 2012	India	Prospective non-randomized	PSU and DGC	51	TUNEL	DFI, post-selection concentration and Post-selection progressively motility	Normozoospermic (n=11) Oligozoospermic(n=20) Teratozoopsermic (n=20)
Zhang et al., 2011	China	Prospective non-randomized	PSU and DGC	12	SCD	DFI	Normozoopsermic
Meitei et al., 2021	India	Prospective non-randomized	PSU and DGC	15	TUNEL	DFI	Normozoopsermic
Oguz et al., 2018	Turkey	RCT	PSU and DGC	65	SCD	DFI, post-selection concentration and post-selection progressively motility	Normozoospermic (N = 34) Non-normozoospermic (N = 31)
Ahmad et al., 2009	Pakistan	Prospective non-randomized	PSU and DGC	221	Comet assay	DFI	Normozoospermic(N = 34) Non-normozoospermic (N = 187)
Hardiyanto et al., 2019	Indonesia	Prospective non-randomized	DSU and DGC	9	SCD	DFI	Normozoospermic
Le et al., 2022	Vietnam	Prospective non-randomized	PSU and DGC	63	SCD	DFI and post-selection concentration	^†^Mixed (Normozoospermic and Oligozoospermic)
Ghaleno et al., 2014	Iran	Prospective non-randomized	PSU, DSU and DGC	28	TUNEL	DFI, post-selection concentration and Post-selection progressively motility	^†^Mixed
Enciso et al., 2011	Spain	Prospective non-randomized	PSU and DGC	157	SCD	DFI	^†^Mixed
Lachaud et al., 2004	Spain	Prospective non-randomized	PSU and DGC	7	TUNEL	DFI	Normozoospermic
Matsuura et al., 2010	Japan	Prospective non-randomized	DGC and DGC-PSU	31	SCSA	DFI	^†^Mixed
Jamil et al., 2023	Morocco	Prospective non-randomized	DGC, DSU and DGC-PSU	97	TUNEL	DFI	Normozoospermic
Volpes et al., 2016	Italy	Prospective non-randomized	PSU, DSU, DGC and DGC-PSU	98	SCD	DFI	^†^Mixed
Jackson et al., (30)	Brazil	Prospective non-randomized	PSU, DGC and DGC-PSU	20	SCD	DFI	Normozoospermic
T. Degheidy et al., 2014	Saudi Arabia	Prospective non-randomized	DGC and DGC-MACS	36	TUNEL	DFI, post-selection concentration and Post-selection progressively motility	Non-normozoospermic (oligoasthenoteratospermia)
Hozyen et al., (36)	Egypt	RCT	DGC and DGC-MACS	151	TUNEL	DFI	^†^Mixed
Lee et al., 2009	China	Prospective non-randomized	DGC and DGC-MACS	60	TUNEL	DFI and post-selection progressively motility	Normozoospermic
H. Zhang et al., 2017	China	Prospective non-randomized	DGC and DGC-MACS	16	TUNEL	DFI	Non-normozoospermia (athenoteratozoospermic)
Cakar et al., 2016	Turkey	Prospective non-randomized	PSU,DGC,DGC-MACS and PSU-MACS	20	TUNEL	DFI, post-selection concentration and Post-selection progressively motility	Normozoospermic (N = 10) Non-normozoospermic (N = 10 oligozoospermic)
Bucar et al., 2015	Portugal	Prospective non-randomized	DGC-PSU and MACS-DSU	40	TUNEL	DFI	^†^Mixed (normo-, terato- and athenozoospermic
Nadalini et al., 2014	Italy	Prospective non-randomized	DGC, DGC-PSU and DGC-MACS	25	TUNEL	DFI and post-selection progressively motility	^†^Mixed
Bibi et al., 2023	Saudi Arabia	Prospective non-randomized	PSU, DGC, DGC-PSU and DGC-MACS	385	SCSA	DFI	Non-normozoospermic (Teratozoospermic)
Mateizel et al., 2024	Belgium	Prospective non-randomized	PSU, DGC and MACS	52	TUNEL	DFI, post-selection concentration and Post-selection progressively motility	^†^Mixed
Chi et al., (13)	Korea	Prospective non-randomized	DGC, MACS and DGC-MACS	29	SCD	DFI	Normozoospermic
M Tavalaee et al., (12)	Iran	Prospective non-randomized	DGC, MACS, DGC-MACS and MACS-DGC	15	TUNEL	DFI	^†^Mixed
T.S.Berteli et al., 2017	Brazil	Prospective non-randomized	DGC, MACS, DGC-MACS and MACS-DGC	15	TUNEL	DFI, post-selection concentration and Post-selection progressively motility	^†^Mixed

*Study conducted two independent experimental phases using different semen samples and was treated as two separate comparisons in this network meta-analysis.

† Mixed = studies that include both normozoospermic and non-normozoospermic samples without reporting sperate outcomes for each semen-quality category.

DGC, density gradient centrifugation; PSU, Pellet Swim-Up; DSU, Direct Swim-Up; DGC-PSU, Swim-Up after DGC (treated as PSU in analysis); MACS, magnetic-activated cell sorting; MACS-DGC, DGC-MACS, PSU-MACS, MACS-WSU, sequential combination methods; MFSS, Microfluidic sperm sorting; RCT, randomized controlled trial; TUNEL, Terminal deoxynucleotidyl transferase dUTP nick end labeling; SCD, sperm chromatin dispersion; SCSA, sperm chromatin structure assay; DFI, DNA fragmentation index.

**Figure 2:**
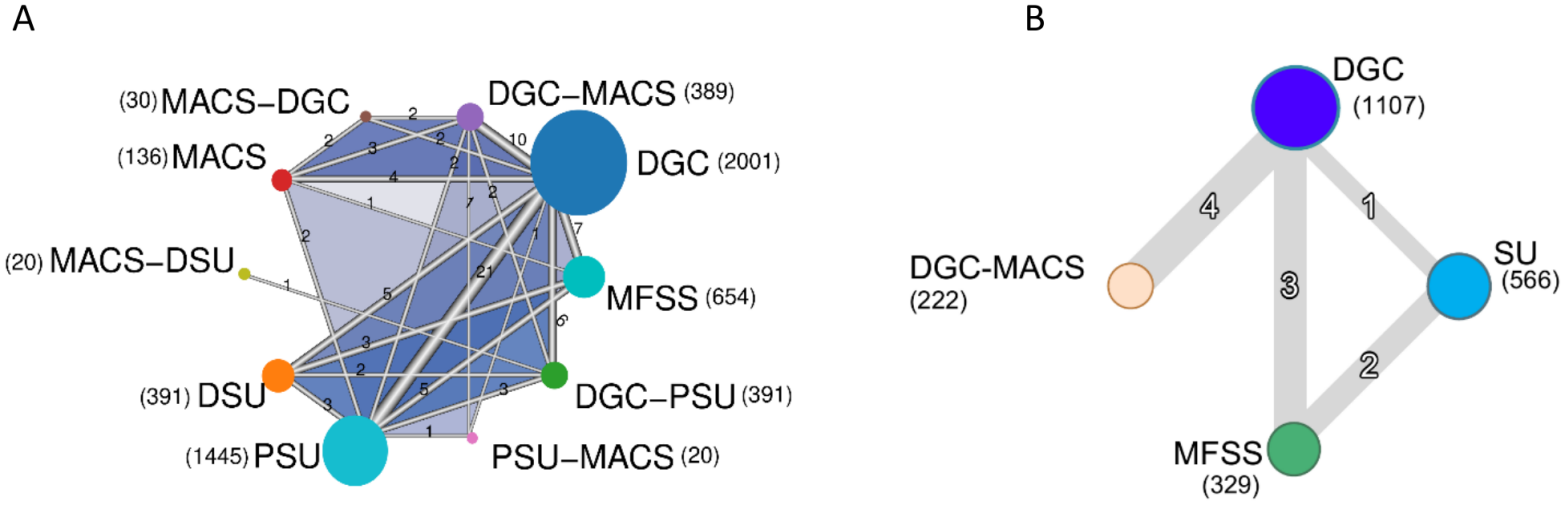
Network plots of sperm preparation techniques for DNA fragmentation and clinical pregnancy outcomes. **(A)** Network plot for the network meta-analysis of sperm DNA fragmentation index (DFI). Each node represents a sperm preparation technique; the size of the node and numbers next to it correspond to the total number of semen samples processed using that technique across all included studies. Lines connecting nodes indicate direct head-to-head comparisons. The numbers displayed on each line represent the total number of direct comparisons between the two connected techniques. **(B)** Network plot for the network meta-analysis of clinical pregnancy rate. Each node represents a sperm preparation technique. Node size and the numbers adjacent to each node indicate the total numbers of couples treated with technique across all included randomized controlled trials. Lines connecting nodes represent direct comparisons between techniques, and the numbers displayed on each line represent the total number of studies contributing to each comparison. DGC, density gradient centrifugation; PSU, Pellet Swim-Up; DSU, Direct Swim-Up; DGC-PSU, Swim-Up after DGC; MACS, magnetic-activated cell sorting; MACS-DGC, DGC-MACS, PSU-MACS, MACS-WSU, sequential combination methods; MFSS, Microfluidic sperm sorting; DGC+MACS, combination of MACS with DGC (non-sequential).

Among the included studies, 21 evaluated traditional sperm preparation techniques. Thirteen studies investigated MACS, alone or in combination with traditional technique, and 9 evaluated MFSS. Overlap between MFSS and MACS comparison groups occurred, as one study compared MACS with MFSS (15) (**Figure 2**).

### 3.2 Characteristics of included studies for clinical outcomes

A total of 15 RCTs were included in the final analysis of clinical outcomes following study selection (**Table 2**; **Supplementary Figure 1**). Among these studies, 13 reported clinical pregnancy rate (23, 35–37, 39–47), 9 reported live birth rates (23, 38, 40–43, 45–47), 8 reported miscarriage rates (23, 36, 39–41, 43, 46, 47), and 4 reported the fertilization rates (35, 43, 44, 48). The sperm preparation techniques evaluated included DGC, SU, combinations of traditional techniques with MACS, and MFSS.

**Table 2:** General characteristics of the randomized controlled trails included in the clinical outcomes analyses.

Study	Country	Sperm preparation techniques	outcomes	ART treatment
Dodson et al., (37)	United States	DGC and SU	Clinical pregnancy (per cycle)	IUI
Carrell et al., (38)	United States	DGC and SU	Clinical miscarriage rate and live birth rate (per cycle)	IUI
Troya et al., (39)	Peru	DGC and combination of MACS and DGC	Clinical pregnancy and clinical miscarriage rate	ICSI
Ozaltin et al., (40)	Turkey	DGC and MFSS (Fertile chip)	Clinical pregnancy, clinical miscarriage rate and live birth rate	ICSI
Ravina et al., (41)	Spain	DGC and combination of MACS and DGC	Clinical pregnancy, clinical miscarriage rate and live birth rate	IUI
Romany et al., (42)	Spain	SU and MACS +SU	Clinical pregnancy and live birth rate	ICSI
Mei et al., (43)	China	DGC-SU and DGC-SU + MACS	Fertilization rate, clinical pregnancy and live birth rate	ICSI
Anbari et al., (44)	Iran	SU and MFSS	Fertilization rate, implantation rate and clinical pregnancy rate	ICSI
Hozyen et al., (36)	Egypt	DGC and combination of MACS and DGC	Clinical pregnancy, implantation rate and clinical miscarriage rate	ICSI
Godiwala et al., (23)	United States	DGC and MFSS (Fertile chip)	Clinical pregnancy, clinical miscarriage rate and live birth rate	ICSI
Yetkinel et al., (45)	Turkey	SU and MFSS (Fertile chip)	Clinical pregnancy and live birth rate	ICSI
Duong et al., (46)	Vietnam	DGC and SU	Clinical pregnancy, miscarriage rate and live birth rate	IUI
Yildiz et al., (35)	Turkey	DGC and MFSS (Fertile chip)	Clinical pregnancy and fertilization rate	ICSI
Ziarati et al., (47)	Iran	DGC and combination of MACS and DGC	Clinical pregnancy and live birth rate	ICSI

DG C, density gradient centrifugation; SU, Swim-Up; DGC-SU, Swim-Up after DGC; MACS, magnetic-activated cell sorting; MFSS, Microfluidic sperm sorting; IUI, Intrauterine Insemination ICSI, Intracytoplasmic Sperm Injection.

### 3.3 Risk of bias assessment

For DFI comparison, based on the NOS scores, most studies achieved a total score of 8 and were judged to be at low risk of bias, while three studies scored 7 and were considered to have moderate risk. No study was classified as high risk (**Supplementary Table 2**). The two RCTs included in the DFI analysis were assessed using the RoB 2 tool. One was judged to have low risk of bias and the other as having some concerns.

For clinical outcomes (clinical pregnancy, miscarriage and live birth), as these outcomes were derived from the same randomized process and follow-up, the risk of bias assessment was identical across clinical outcomes. Nine out of fifteen RCTs were judged with some concerns, primarily due to no insufficient information about allocation concealment, while the remaining RCTs were judged to be at low risk of bias (**Supplementary Figure 2**).

### 3.4 Quality of evidence

Using the CINeMA framework, the confidence in the evidence for DFI outcomes ranged from very low to moderate across network comparisons (**Supplementary Table 3**). Eleven key comparisons were selected for focused presentation in **Table 3**. Heterogeneity and incoherence domains presented some or major concerns, particularly for comparisons informed by indirect evidence, which led to downgrading of confidence in several estimates.

**Table 3:** GRADE/CINeMA summary table for key comparisons evaluating on sperm DNA fragmentation and clinical pregnancy outcome.

Outcome	Comparisons	Number of studies	Within-study bias	Reporting bias	Indirectness	Imprecision	Heterogeneity	Incoherence	NMA quality
Sperm DNA fragmentation	MFSS vs DGC	7	⊕⊕⊕⊕	⊕⊕⊕⊙	⊕⊕⊕⊕	⊕⊕⊕⊕	⊕⊕⊕⊕	⊕⊕⊕⊕	Moderate
	MFSS vs PSU	5	⊕⊕⊕⊕	⊕⊕⊕⊙	⊕⊕⊕⊕	⊕⊕⊕⊕	⊕⊕⊕⊙	⊕⊕⊕⊕	Low
	MFSS vs DGC-PSU	Indirect*	⊕⊕⊕⊕	⊕⊕⊕⊙	⊕⊕⊕⊕	⊕⊕⊕⊙	⊕⊕⊕⊙	⊕⊕⊙⊙	Very low
	MFSS vs MACS	1	⊕⊕⊕⊕	⊕⊕⊕⊙	⊕⊕⊕⊕	⊕⊕⊕⊙	⊕⊕⊕⊙	⊕⊕⊕⊕	Low
	MFSS vs DGC-MACS	Indirect*	⊕⊕⊕⊕	⊕⊕⊕⊙	⊕⊕⊕⊕	⊕⊕⊕⊕	⊕⊕⊙⊙	⊕⊕⊙⊙	Very low
	MACS-DGC vs DGC	2	⊕⊕⊕⊕	⊕⊕⊕⊙	⊕⊕⊕⊕	⊕⊕⊕⊕	⊕⊕⊙⊙	⊕⊕⊕⊕	Low
	MACS vs DGC	4	⊕⊕⊕⊕	⊕⊕⊕⊙	⊕⊕⊕⊕	⊕⊕⊕⊙	⊕⊕⊕⊙	⊕⊕⊕⊙	Low
	DGC-MACS vs DGC	10	⊕⊕⊕⊕	⊕⊕⊕⊙	⊕⊕⊕⊕	⊕⊕⊕⊕	⊕⊕⊙⊙	⊕⊕⊕⊙	Low
	DSU vs DGC	6	⊕⊕⊕⊕	⊕⊕⊕⊙	⊕⊕⊕⊕	⊕⊕⊕⊕	⊕⊕⊙⊙	⊕⊕⊕⊕	Low
	PSU vs DGC	21	⊕⊕⊕⊕	⊕⊕⊕⊙	⊕⊕⊕⊕	⊕⊕⊕⊕	⊕⊕⊙⊙	⊕⊕⊕⊕	Low
	DGC-PSU vs DGC	6	⊕⊕⊕⊕	⊕⊕⊕⊙	⊕⊕⊕⊕	⊕⊕⊕⊕	⊕⊕⊙⊙	⊕⊕⊕⊕	Low
Clinical pregnancy outcome	DGC vs combination of MACS and DGC	4	⊕⊕⊕⊙	⊕⊕⊕⊙	⊕⊕⊕⊙	⊕⊕⊕⊕	⊕⊕⊙⊙	⊕⊕⊕⊕	Low
	MFSS vs DGC	3	⊕⊕⊕⊙	⊕⊕⊕⊙	⊕⊕⊕⊙	⊕⊕⊙⊙	⊕⊕⊕⊕	⊕⊕⊕⊕	Low
	DGC vs SU	1	⊕⊕⊕⊕	⊕⊕⊕⊙	⊕⊕⊕⊙	⊕⊕⊙⊙	⊕⊕⊕⊕	⊕⊕⊕⊕	Very low
	MFSS vs SU	2	⊕⊕⊕⊕	⊕⊕⊕⊙	⊕⊕⊕⊙	⊕⊕⊙⊙	⊕⊕⊕⊕	⊕⊕⊕⊕	Low
	MFSS vs combination of MACS and DGC	0	⊕⊕⊕⊙	⊕⊕⊕⊙	⊕⊕⊕⊙	⊕⊕⊕⊕	⊕⊕⊙⊙	⊕⊕⊕⊕	Very low
	combination of MACS and DGC vs SU	0	⊕⊕⊕⊕	⊕⊕⊕⊙	⊕⊕⊕⊙	⊕⊕⊕⊕	⊕⊕⊕⊕	⊕⊕⊕⊕	Low

This table summarize the certainty of evidence for key comparisons for DNA fragmentation and clinical pregnancy outcome using the GRADE framework. Each domain is rated according to CINeMA’s judgements (no concerns, some concerns, major concerns) and translated into GRADE symbols: ⊕⊕⊕⊕ — no concerns (no downgrade), ⊕⊕⊕⊙ —some concerns (downgrade one level) and ⊕⊕⊙⊙ —major concerns (downgraded two levels).

*Indirect means when the network estimate is derived exclusively from indirect evidence. The final NMA quality reflects CINeMA’s overall confidence in the network estimate.

DGC, density gradient centrifugation; PSU, Pellet Swim-Up; DSU, Direct Swim-Up; DGC-PSU, Swim-Up after DGC (treated as PSU in analysis); MACS, magnetic-activated cell sorting; MACS-DGC, DGC-MACS, PSU-MACS, MACS-WSU, sequential combination methods; MFSS, Microfluidic sperm sorting; GRADE, Grading of Recommendations Assessment, Development and Evaluation; CINeMA, Confidence in Network Meta-Analysis.

For clinical pregnancy outcome, the confidence in the evidence ranged from low to very low (**Table 3**). Major concerns were primarily related to heterogeneity and imprecision, which contributed to the downgrading of confidence ratings.

### 3.5 Primary outcome: sperm DNA fragmentation

#### 3.5.1 Pairwise meta-analysis results

Pairwise meta-analyses were conducted for eight direct comparisons: DGC-PSU vs PSU, MFSS vs PSU, DSU vs DGC, MFSS vs DGC, DGC-MACS vs DGC, PSU vs DGC, DGC-PSU vs DGC and DSU vs PSU. (**Table 4**; **Supplementary Figure 3**).

**Table 4:** Pairwise and network meta-analysis of sperm DNA fragmentation using different preparation techniques.

Outcome	Comparisons	Pairwise meta-analysis		Network meta-analysis SMD (95% CI)
		No. of studies	SMD (95% CI)	
Sperm DNA fragmentation	DGC as a reference			
	PSU	21	─0.97 (─1.83, ─0.12)	─0.81 (─1.28, ─0.34)
	DSU	5	─1.37 (─2.63, ─0.12)	─1.38 (─2.20, ─0.55)
	DGC-PSU	6	─0.94 (─1.68, ─0.21)	─1.27 (─2.09, ─0.45)
	DGC-MACS	10	─0.61 (─0.98, ─0.23)	─0.92 (─1.60, ─0.25)
	MFSS	5	─3.53 (─9.72, 2.65)	─2.64 (─3.53, ─1.75)
	MACS	*NA	*NA	─0.59 (─1.51, 0.33)
	MACS-DGC	*NA	*NA	─1.53 (─2.93, ─0.12)
	MACS-DSU	*NA	*NA	─1.20 (─3.63, 1.22)
	PSU-MACS	*NA	*NA	─1.01 (─2.90,0.87)
	PSU as a reference			
	DSU	3	─0.25 (─4.17,3.67)	0.51 (─5.26, 6.28)
	DGC-PSU	3	─0.59 (─2.84, 1.67)	1.02 (─4.57, 6.62)
	MFSS	4	─12.66 (─50.21, 24.90)	─2.06 (─3.26, ─0.86)
	DSU as a reference			
	MFSS	2	*NA	─1.27 (─2.32, ─0.21)

*NA indicates comparisons for which direct evidence was unavailable or not performed. DGC, density gradient centrifugation; PSU, Pellet Swim-Up; DSU, Direct Swim-Up; DGC-PSU, Swim-Up after DGC (treated as PSU in analysis); MACS, magnetic-activated cell sorting; MACS-DGC, DGC-MACS, PSU-MACS, MACS-WSU, sequential combination methods; MFSS, Microfluidic sperm sorting; MD, mean difference; CI, confidential interval.

Direct comparisons showed that MFSS was associated with lower DFI compared with DGC (SMD −3.53; 95% CI −9.72 to 2.65) and PSU (SMD −12.66; 95% CI −50.21 to 24.90); but with no statistically significant. PSU, DSU, DGC-PSU and DGC-MACS were also associated with significantly lower DFI compared with DGC. Comparisons of DGC-PSU and DSU versus PSU suggested a slightly greater reduction in DFI, but these differences did not reach statistical significance.

#### 3.5.2 Network meta-analysis

Random-effects network meta-analysis showed that several sperm preparation techniques were associated with significantly lower DFI compared with DGC (**Table 4**). These included MFSS (SMD −2.64; 95%CI: −3.54 to −1.75), MACS-DGC (SMD −1.53; 95%CI: −2.93 to −0.11), DGC-PSU (SMD−1.27; 95%CI: −2.09 to −0.45), DSU (SMD−1.38; 95%CI: −2.20 to −0.55), PSU (SMD−0.81; 95%CI: −1.28 to −0.34) and DGC-MACS (SMD −0.92; 95%CI: −1.60 to −0.24). In contrast, others were not significant.

SUCRA ranking suggested that MFSS (P = 0.97) tended to rank highest for DFI reduction, followed by MACS-DGC (P = 0.67). However, given the substantial heterogeneity observed, these rankings should be interpreted with caution.

#### 3.5.3 Publication bias

Comparison adjusted funnel plot and Egger’s regression test (**Supplementary Figure 4**) didn’t show statistically significant asymmetry (*p* = 0.33), suggesting no clear evidence of publication bias.

#### 3.5.4 Transitivity and inconsistency

To evaluate the transitivity assumption, we compared the distribution of key clinical and methodological effect modifiers across treatment comparisons (**Supplementary Table S6**). The proportion of studies including normozoospermic, non-normozoospermic, and mixed populations was broadly comparable across treatment nodes, with most studies involving mixed or normozoospermic populations. Additionally, the distribution of DNA fragmentation methods was generally consistent across interventions, with TUNEL and SCD being the most frequently used assays. However, some treatment comparisons were limited by numbers of studies, which may introduce uncertainty in indirect estimates and should be considered when interpreting the results (**Supplementary Table 6**).

Violin and box plots (**Supplementary Figure 5**) were also generated to compare the distributions of key effect modifiers—assay type and sperm quality—across interventions. Overall, both modifiers are distributed similarly across most interventions, supporting the transitivity. Exceptions were observed for MACS-DGC and PSU-MACS, likely reflecting the limited number of studies evaluating these interventions, rather than the true systematic difference in assay and sperm-quality selection.

High heterogeneity was observed across the network (τ^2^ = 1.26, τ =1.12; *I^2^ = 96.1%*). Global inconsistency testing indicated (**Supplementary Table 4**) significant heterogeneity and inconsistency (Q = 1299.82, df=51, *p<0.001*). However, node-splitting analyses showed that inconsistency was primarily confined to the DGC-MACS vs DGC and MACS vs PSU comparisons, whereas all other contrasts demonstrated good agreement between direct and indirect evidence. The design by treatment interaction model revealed significant within-design heterogeneity for comparisons of DGC vs PSU, DGC vs MFSS, and DGC vs DGC-MACS comparisons. All between-design detachment analyses remained significant, indicating that removal of single design did not resolve the global inconsistency. (**Supplementary Table 5**).

#### 3.5.5 Subgroup analysis by assay type

Subgroup analyses were performed to explore potential source of the high heterogeneity. Five assays were used across included studies: TUNEL (19 studies), SCD (17 studies), SCSA (3 studies), comet assay (2 studies) and toluidine blue (1 study). Due to the limited number of studies employing SCSA, comet assay and toluidine blue, these were not included in subgroup network meta-analyses.

In both the TUNEL and SCD subgroups, MFSS, DGC-PSU, PSU and DSU were associated with significantly lower DFI compared with DGC. In the TUNEL subgroup, MACS-DGC and DGC-MACS were also associated with significantly reduced DFI (SMD −1.43; 95%CI−2.74 to −0.11; and SMD −0.83; 95%CI: −1.53 to −0.12);, whereas MACS alone did not show a significant effect. Similar treatment effects were observed in both the TUNEL and SCD subgroups.

SUCRA ranking indicated that MFSS tended to rank highest for DFI reduction in both the TUNEL (P = 0.91) and SCD (P = 0.98) subgroups. DGC-PSU ranked second in the TUNEL subgroup(P = 0.64), whereas DGC-MACS ranked second in the SCD subgroup (P = 0.61). However, substantial heterogeneity remained high across subgroups (TUNEL *I^2^ = 92.8%*, SCD *I^2^ = 95.9%*), indicating that stratification by assay type did not fully explain the observed variability.

#### 3.5.6 Subgroup analysis by semen quality

Eighteen studies (N = 479) were included in the normozoospermia subgroup network meta-analysis. Heterogeneity persisted (*I^2^ = 93.4%*), and random-effects models were applied. Compared with DGC, MFSS and PSU were associated with significantly lower DFI (SMD −1.78; 95% CI−3.03 to −0.53; and SMD−1.03; 95% CI −1.72 to −0.34; respectively). SUCRA ranking (**Supplementary Figure 6**) indicated MFSS was the most effective technique(P = 0.84), followed by PSU-MACS (P = 0.64) and DSU(P = 0.59).

Nine studies (N = 757) were selected in the non-normozoospermia subgroup network meta-analysis. Comprising oligozoospermic, teratozoospermic, asthenoteratozoospermic, and unspecified abnormal semen samples. Heterogeneity remained high (*I^2^ = 87.5%*). When compared with DGC, MFSS and DGC-MACS significantly reduced DFI (SMD −1.54; 95% CI−2.24 to −0.84; and SMD −0.75; 95% CI−1.44 to −0.06; respectively). SUCRA analysis (**Supplementary Figure 6**) ranked MFSS highest (P = 0.94), followed by MACS (P = 0.70) and DSU (P = 0.67).

#### 3.5.7 Meta regression

Due to more than 30% missing data of male age, we only included baseline DFI, raw semen concentration, total motility and assay type for univariate meta-regression analyses. None of these covariates significantly explained the between-study heterogeneity, as the estimated between study variance remained unchanged across all models (τ^2^ =1.29, *I^2^ =* 95.6%).

#### 3.5.8 Sensitivity analysis

Design-based sensitivity analysis showed that the network meta-analysis results remained unchanged after exclusion of the two RCTs (τ^2^ =1.18, *I^2^ =* 95.8%).

Influence analysis (leave-one-out interations) based on Cochran’s Q statistic identified three studies (Miraseni et al., 2022, Ahmad et al., 2009, and Oguz et al., 2018) that contributed disproportionately to the global heterogeneity. Sequential removal of these studies reduced heterogeneity from 96.6% to 93.9%, and further to 93.3% after exclusion of all three studies. The high residual heterogeneity suggests that the observed variability was not driven by individual studies.

Overall, the network meta-analyses results remained consistent across both design-based and influenced-based sensitivity analyses.

### 3.6 Secondary outcomes: clinical outcomes

Clinical pregnancy was the only clinical outcomes that analyzed using network meta-analysis. Due to the limited numbers of studies, distinctions between PSU and DSU, as well as the sequential order of DGC and MACS, were not analyzed separately and were therefore not incorporated into the network. In addition, only one study (42) compares SU with SU-MACS, and one study (43) compared between DGC-SU with DGC-SU-MACS, these interventions were not included as independent nodes in the network.

**Figure 3:**
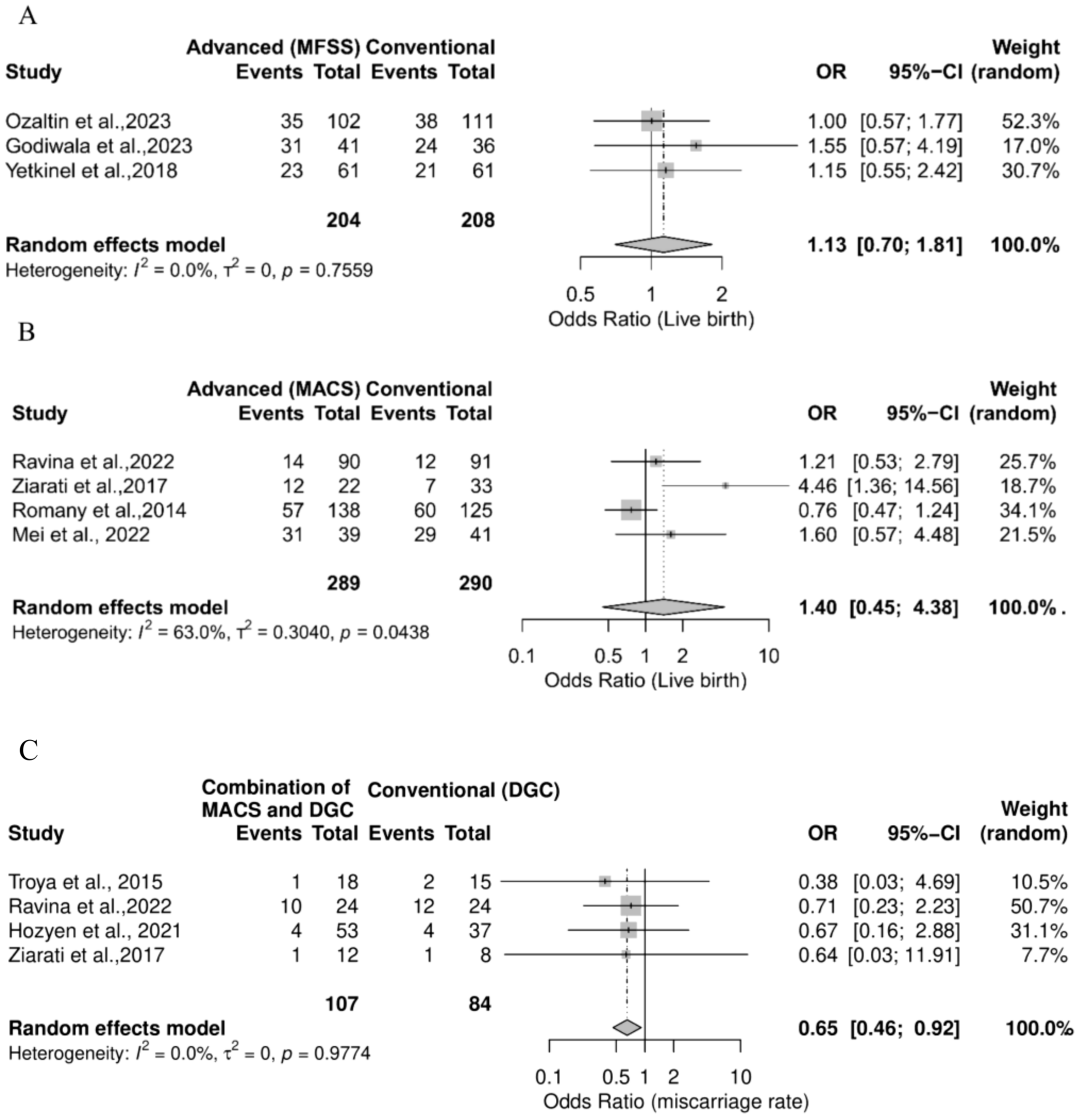
Forest plots for pairwise meta-analysis of live birth rate and miscarriage rate. **(A)** Forest plot of stratified pairwise meta-analysis comparing the live birth rate between microfluidic sperm sorting (MFSS) and conventional sperm preparation techniques. Two of the three include studies compared between MFSS with density gradient centrifugation (DGC) (Ozaltin et al., (40) and Godiwala et al., (23), while one study compared MFSS with swim-up (SU) (Yetkinel et al., (45). **(B)** Forest plot of stratified pairwise meta-analysis comparing magnetic-activated cell sorting (MACS) based advanced sperm preparation with conventional sperm preparation. Conventional sperm preparation techniques, including DGC, SU and DGC-SU, were pooled into a single comparator group, whereas combinations of conventional techniques with MACS were grouped as advanced sperm preparation techniques. **(C)** Forest plot of pairwise meta-analysis comparing DGC alone with the combination of MACS and DGC.

Consequently, the clinical pregnancy network meta-analysis was restricted to four sperm preparation techniques: DGC, SU, combination of MACS and DGC, and MFSS. Among the 13 studies reporting clinical pregnancy outcomes, 10 were included in the network meta-analysis. Compared with DGC, combination of MACS and DGC was associated with significantly higher clinical pregnancy rate (OR 1.93; 95% CI 1.23 to 3.02) (**Figure 3**). Whereas SU and MFSS showed no significant difference relative to DGC. No significant heterogeneity or inconsistency was detected within the network (*I^2^* = 24.8%; 95% CI 0.0% to 65.8%).

For live birth rate, three studies comparing MFSS with conventional sperm preparation methods (DGC and SU) were pooled in a stratified pairwise meta-analysis, MFSS was associated with a higher live birth rate compare with conventional methods; however, this difference was not statistically significant (OR 1.15; 95% CI 0.55 to 2.42; *I^2^* = 0%) (**Figure 3**). Four studies comparing MACS based advanced sperm preparation with conventional sperm preparation were included in a separate stratified pairwise meta-analysis. Although the pooled estimate favored MACS-based methods, the association was not statistically significant and moderate heterogeneity was observed (OR 1.40; 95% CI 0.45 to 4.38; *I^2^* = 63%) (**Figure 3**).

For miscarriage rate, four studies comparing DGC with the combination of MACS and DGC were pooled in a pairwise meta-analysis. The pooled results indicated that the addition of MACS to DGC was associated with a significantly lower miscarriage rate compared with DGC alone (OR 0.65; 95% CI 0.46 to 0.91); (**Figure 3**), although substantial heterogeneity was observed (*I*^2^ = 84.7%). Two studies compared miscarriage rates between MFSS and DGC, but their findings were inconsistent. A lower miscarriage rate with MFSS compared with DGC was reported in one study (23), whereas another study reported a higher miscarriage rate with MFSS (40).

## 4 Discussion

Optimizing sperm selection is critical for improving ART outcomes, particularly in the context of increasing recognition of sperm DNA integrity as a key determinant of reproductive success (49). In this network meta-analysis of 42 studies, we compared multiple sperm preparation techniques, however, the very high and persistent heterogeneity across studies (*I*^2^ = 96.1%), together with inconsistencies in the existing literature, substantially limits the interpretability of the pooled results. Therefore, our findings primarily reflect the variability and methodological limitations of the current evidence base, rather than establishing the comparative effectiveness of different sperm preparation techniques. While some advanced techniques (e.g. MFSS and the combination of MACS with DGC) appeared to be associated with greater reductions in DNA fragmentation, these findings should be interpreted with considerable caution given the high heterogeneity.

A key limitation of the present analysis is the extremely high heterogeneity observed across studies, which persisted despite multiple analytical approaches, including the use of standardized mean difference to account for differences in DFI measurement, subgroup analyses stratified by assay type and semen quality, and meta-regression adjusting for baseline DFI and raw semen parameters. The inability to explain heterogeneity through commonly reported study-level variables suggests that unmeasured or inconsistently reported factors may play a substantial role. In particular, more than 30% of included studies did not report male age. Given that sperm DNA fragmentation has increasingly been linked to advanced paternal age (50), the absence of these data limited assessment of paternal age as a potential source of heterogeneity and residual confounding within the network. Sensitivity analyses further indicated that, although certain studies contributed disproportionately to heterogeneity, their exclusion did not meaningfully reduce it, indicating that the variability is not driven solely by outliers’ studies.

Importantly, several assumptions underlying network meta-analysis may not have been fully met. The inclusion of heterogeneous populations, including normozoospermic men, patients with abnormal semen parameters, and sperm donors, raises concerns regarding the transitivity assumption, as these groups may differ in baseline risk and in their response to sperm preparation techniques. In addition, the inclusion of both randomized and non-randomized studies within the same network may introduce bias and affect the validity of the findings, given the greater susceptibility of observation studies to selection bias and confounding.

Furthermore, sperm DNA fragmentation was assessed using multiple non-equivalent assays (TUNEL, SCSA, SCD and comet), each capturing different aspects of DNA damage. Pooling these methods may introduce systematic measurement bias, as these assays are not directly comparable and differ in sensitivity. Previous studies have reported only moderate correlations between these assays, further highlighting their limited interchangeability. Although standardization approaches were applied, this does not fully account for inter-assay variability. Subgroup analyses by assay type were planned to explore this source of heterogeneity, however, they were only feasible for TUNEL and SCD based studies due to the limited number of studies using other assays (SCSA and comet assay). Consequently, the potential impact of assay-specific differences on the pooled estimates could not be fully evaluated within this network meta-analysis. Therefore, the findings should be interpreted with caution, as differences between sperm preparation techniques may partly reflect methodological heterogeneity rather than true biological effects, and residual measurement variability cannot be excluded.

Within these limitations, MFSS was frequently among the more effective techniques for reducing DFI, and a similar pattern was observed in subgroup analyses. These results are also supported by several head-to-head studies demonstrating significantly lower DFI in MFSS-selected sperm compared with traditional methods (16–19, 34, 51, 52) and MACS-based approaches (15). However, inconsistent findings in certain subgroups (20)—particularly in the non-normozoospermic group, highlight the potential influence of population characteristics and study design. The enhanced performance of MFSS may be explained by its avoidance of centrifugation, which has been associated with increased ROS generation and potential DNA damage; however, such mechanisms remain hypothetical and were not directly assessed in the included studies (16).

MACS is a widely recommended advanced technique for patients with elevated DFI (12, 13). In the present analysis, MACS alone showed limited effectiveness, whereas its combination with traditional techniques—particularly when applied before DGC (MACS-DGC)—was associated with greater reduction in DFI. This observation may be related to the biology of phosphatidylserine (PS) externalization, which is not restricted to apoptotic sperm (15) but can also occur transiently during capacitation (53). Because DGC may induce capacitation-like changes (54, 55), performing MACS after DGC may theoretically lead to the removal of some viable sperm undergoing physiological membrane remodeling. Whereas applying MACS prior to DGC may more selectively targets truly apoptotic sperm. Nevertheless, these explanations remain speculative and should be interpreted cautiously.

Regarding clinical outcomes, the available evidence remains limited and inconclusive. Although live birth rate is the most clinically relevant outcome in assisted reproduction, it could not be used as the primary endpoint in this study due to the limited number of available studies and the lack of comparable data across sperm preparation techniques. In contrast, clinical pregnancy was more consistently reported and allowed for a more robust network meta-analysis. Nevertheless, the limited evidence on live birth outcomes represents an important limitation of this study, and the findings based on clinical pregnancy should be interpreted with caution, as they may not fully reflect reproductive success.

In addition, although female age was generally reported and appeared comparable between control and intervention groups (**Supplementary Table 7**), ovarian reserve markers such as anti- Müllerian hormone were poorly indicated. Given the strong association between ovarian reserve and ART outcomes (56), the absence of these data limits the ability to fully assess baseline comparability and introduces the potential for residual confounding. This further constrains the interpretation of the pooled clinical results.

No significant advantage was observed for MFSS in terms of clinical pregnancy or live birth rates. While certain comparisons, such as the combination of DGC and MACS, suggested potential improvements in clinical pregnancy rates and lower miscarriage rates compared with DGC alone, these findings were based on a small number of studies and are subject to substantial uncertainty. Overall, the discrepancy between more pronounced effects of techniques like MFSS on DFI reduction and the lack of corresponding improvement in clinical outcomes underscores the uncertain clinical utility of DFI as a surrogate endpoint for ART outcomes.

These findings suggest that sperm DNA fragmentation alone may not be enough to predict reproductive outcomes, which are inherently influenced by multiple paternal and maternal factors, particularly oocyte quality, female age and ovarian reserve. Emerging evidence (57) suggests that female factors may exert a greater influence on embryo development and reproductive outcomes than sperm DNA fragmentation itself, and may therefore act as dominant effect modifiers when evaluating the clinical relevance of DFI. Previous studies (58) have suggested that early developmental impairment associated with sperm DNA fragmentation can possibly be mitigated by the DNA repair capacity of the oocyte. Female age is one of the most crucial factors affecting oocyte quality and may influence the ability of oocytes to repair sperm DNA damage. Consequently, the impact of sperm DNA fragmentation on reproductive outcomes may be more pronounced in women with advanced maternal age or reduced ovarian reserve. However, these female factors were poorly reported in the included studies and could not be adequately accounted for in the present analysis. Therefore, the effect of DNA fragmentation on clinical outcomes likely reflects the interaction between male and female factors, namely the degree of sperm DNA damage and the capacity of oocyte to repair such damage.

## 5 The limitations

This study has several limitations. First, the mixing of study designs—specially the inclusion of observation studies alongside RCTs—may introduce bias. Although many observational studies were rated as low risk of bias via the NOS, this tool may not fully account for residual confounding inherent to non-randomized data; therefore, the overall risk of bias may be underestimated.

Second, substantial heterogeneity was observed across analyses, particularly for DFI outcomes, and persisted despite subgroup analyses by assay type and semen quality. This variability likely reflects procedural differences that are often under-reported, such as differences in MFSS device types, centrifugation protocols, and abstinence period. Regarding publication bias, while no evidence was detected using standardized mean difference, we acknowledge that the interpretation of funnel plots remains limited in the presence of high heterogeneity.

Third, the transitivity assumption may be affected by the methodological diversity across the network. Although our assessment showed similar distribution of sperm quality and assay types across different sperm preparation techniques, the significant global inconsistency suggested potential violations. However, node splitting analyses indicated that inconsistency was driven by two specific comparisons: DGC-MACS vs DGC and MACS vs PSU. In the case of DGC-MACS, both direct and indirect estimates agreed on the direction of effect in DFI reduction. For the remaining comparisons, we found a good agreement between direct and indirect evidence.

Future studies with standardized methodologies are needed to reduce between-study variability and confirm these findings. In addition, more well-designed RCTs comparing advanced sperm preparation techniques with conventional techniques are needed to strengthen the evidence base for clinical outcomes.

## 6 Conclusions

In conclusion, while MFSS appear to be among the most effective techniques at reducing sperm DNA fragmentation, the overall certainty of evidence is low due to substantial and persistent heterogeneity. Furthermore, there is a lack of robust data demonstrating that these biological improvements translate into superior clinical pregnancy or live birth rates. Given these uncertainties, these techniques should be used with caution until large-scale, standardized RCTs confirm their clinical utility.

## Data Availability

The original contributions presented in the study are included in the article/**Supplementary Material**. Further inquiries can be directed to the corresponding author.
