# Supplementary figures and images for: Comparative analysis of sperm preparation techniques on DNA fragmentation and clinical outcomes: a network meta-analysis
Source: Front Endocrinol (Lausanne). 2026 Jul 13;17:1817587. doi: 10.3389/fendo.2026.1817587 (PMC13402121; doi:10.3389/fendo.2026.1817587)

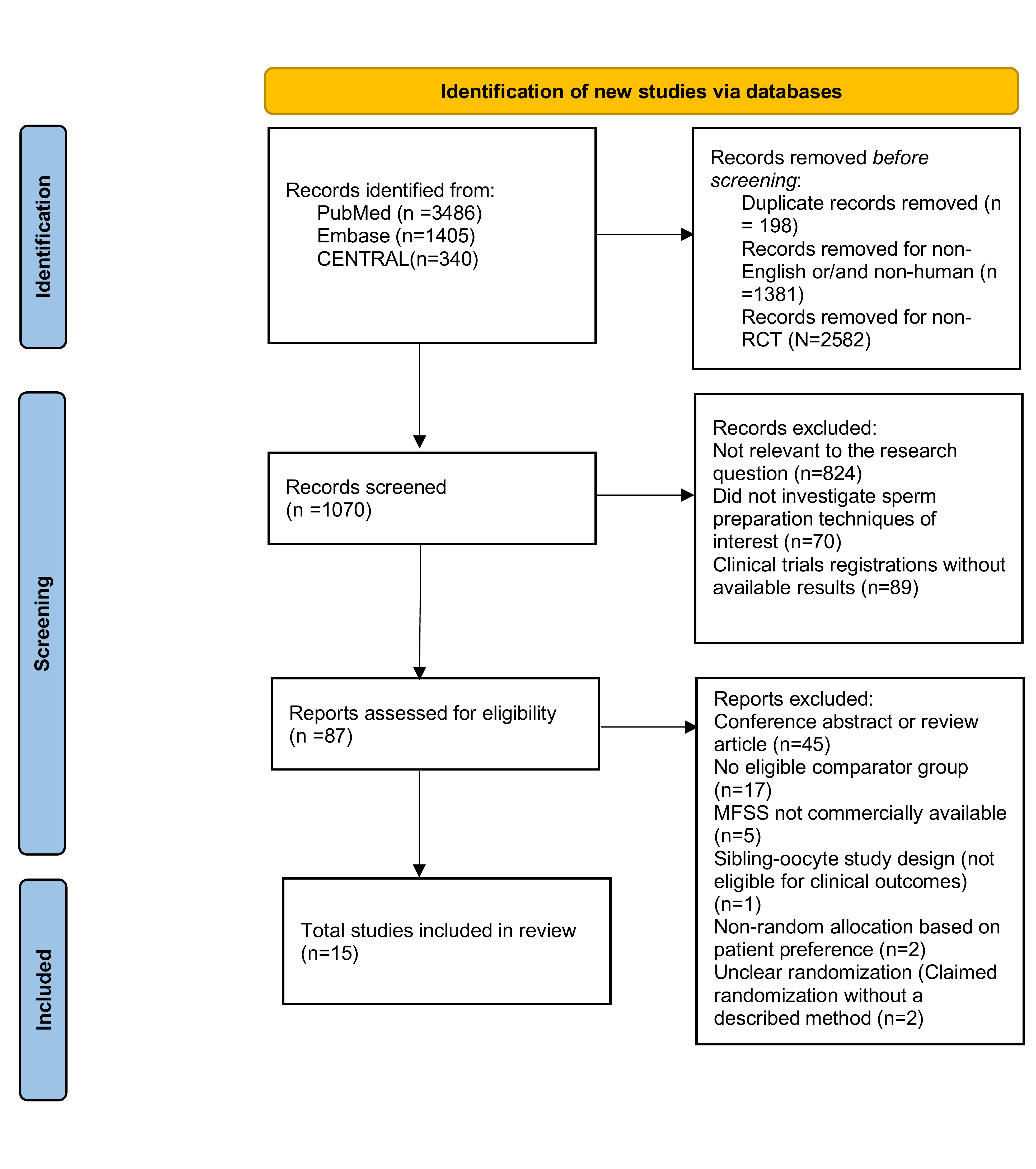

Supplement: Supplementary file 1 [file Image1.tiff]

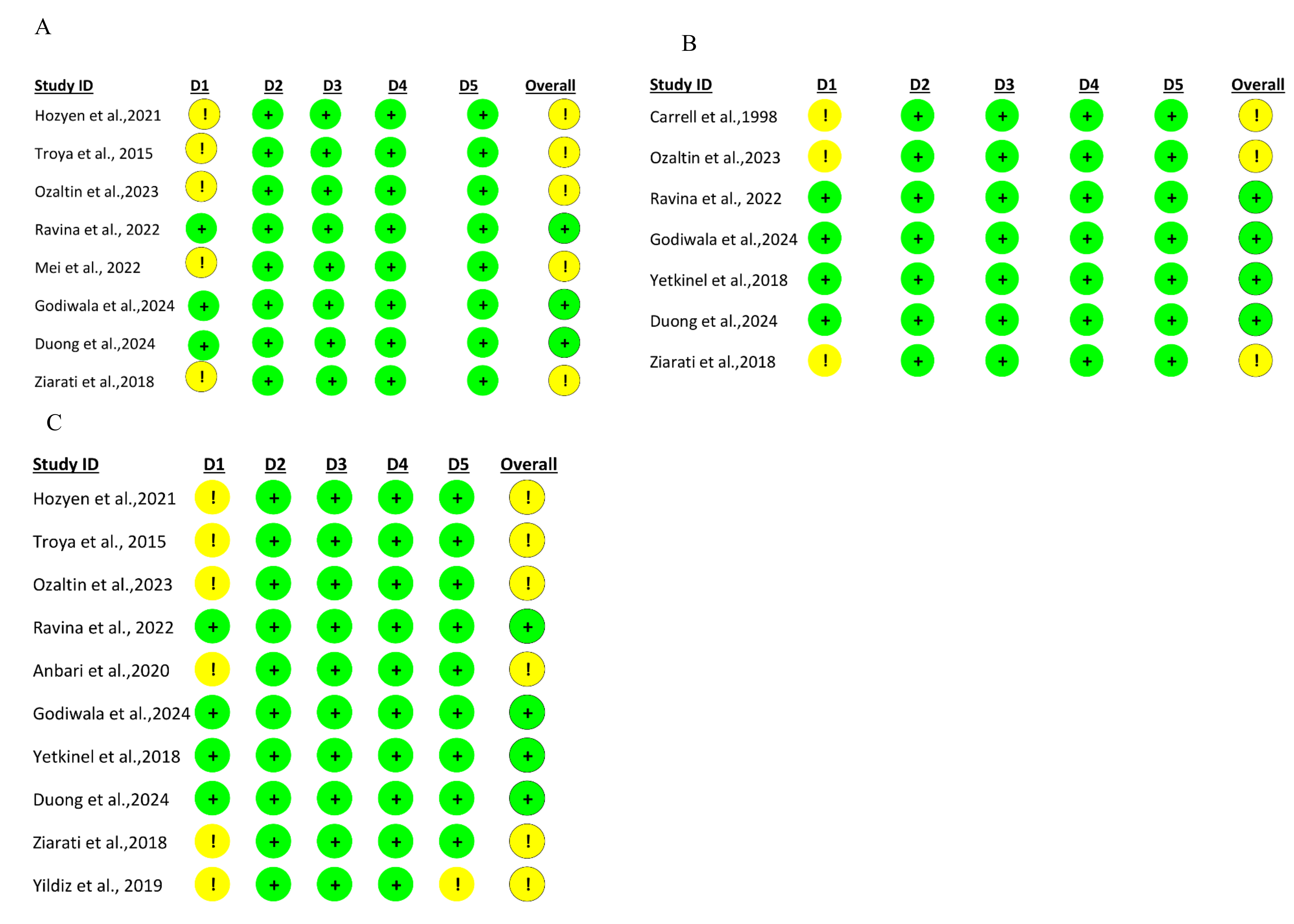

Supplement: Supplementary file 2 [file Image2.tiff]

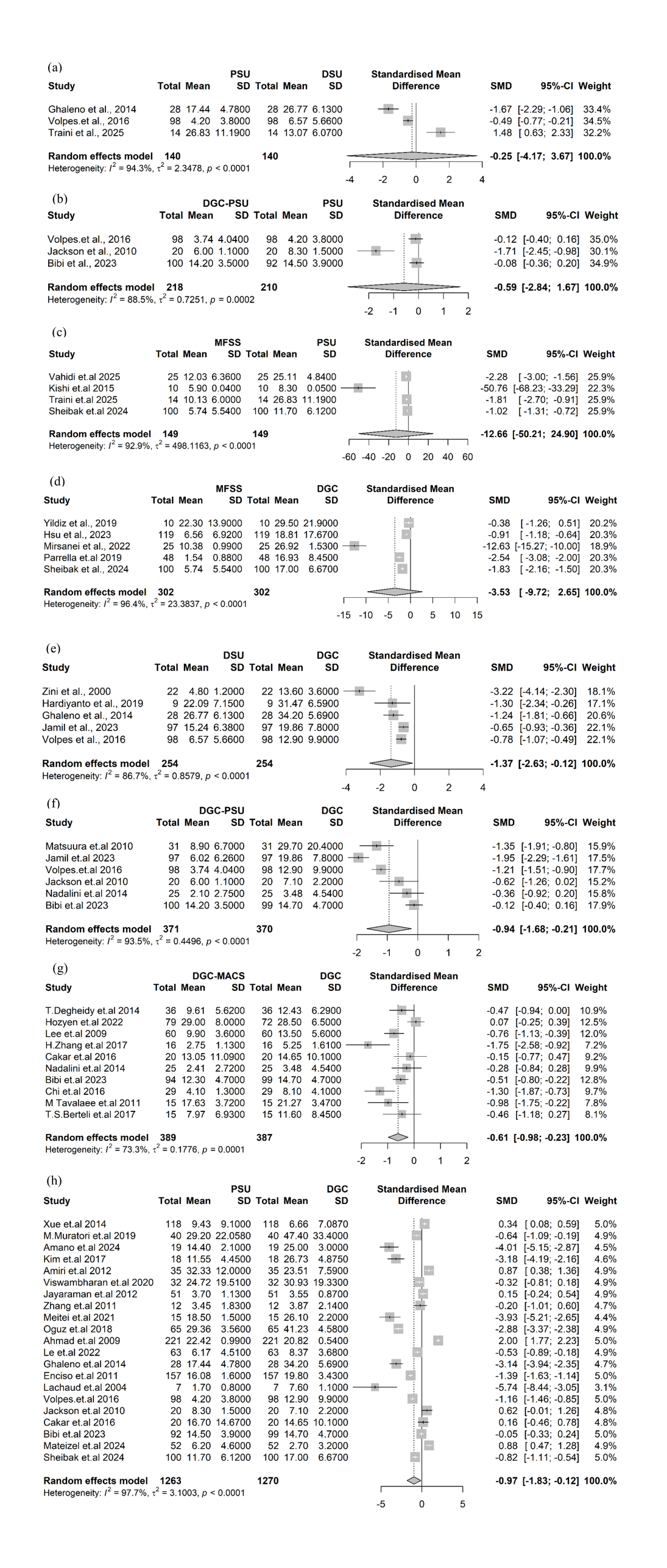

Supplement: Supplementary file 3 [file Image3.tiff]

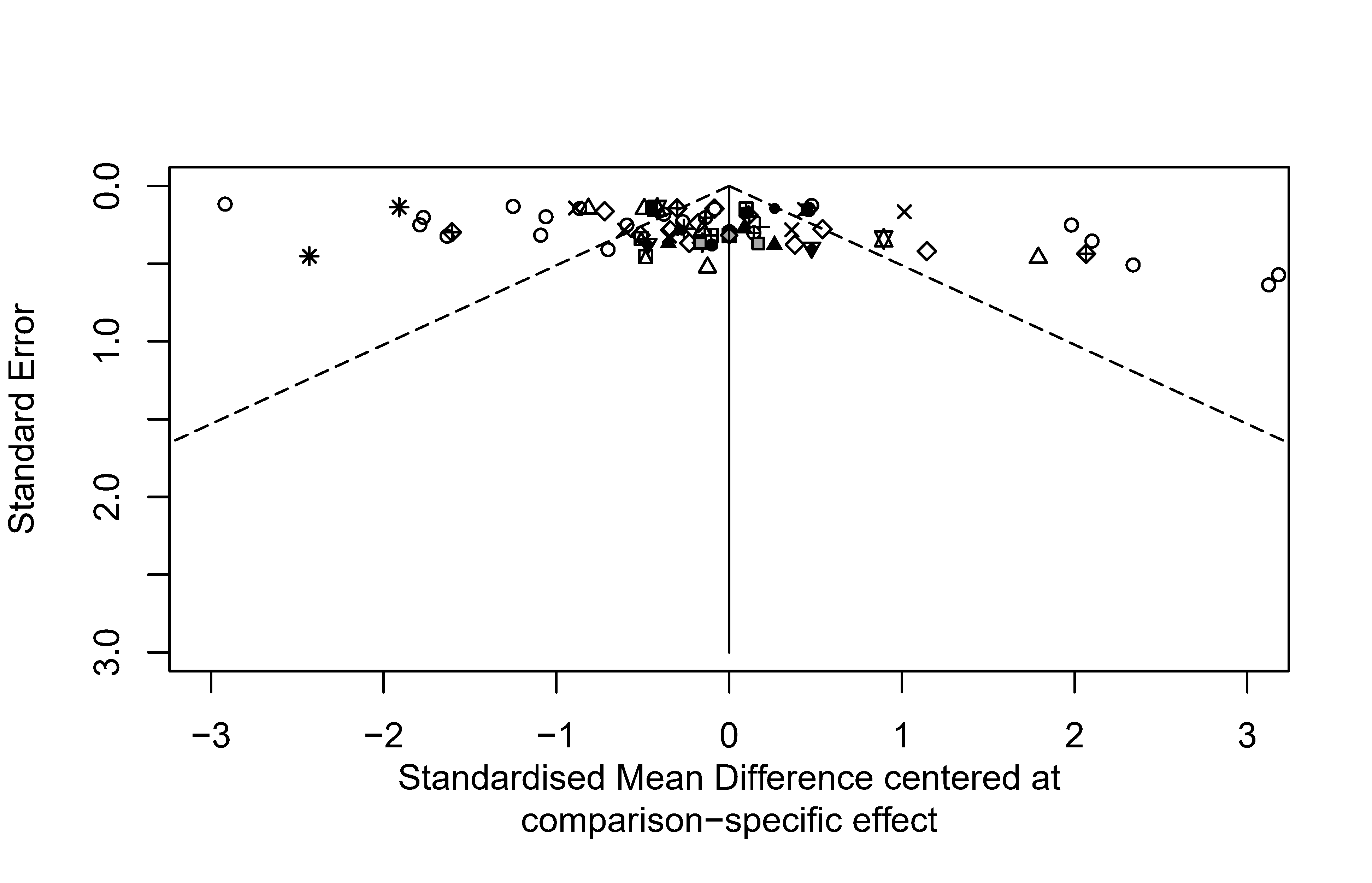

Supplement: Supplementary file 4 [file Image4.tiff]

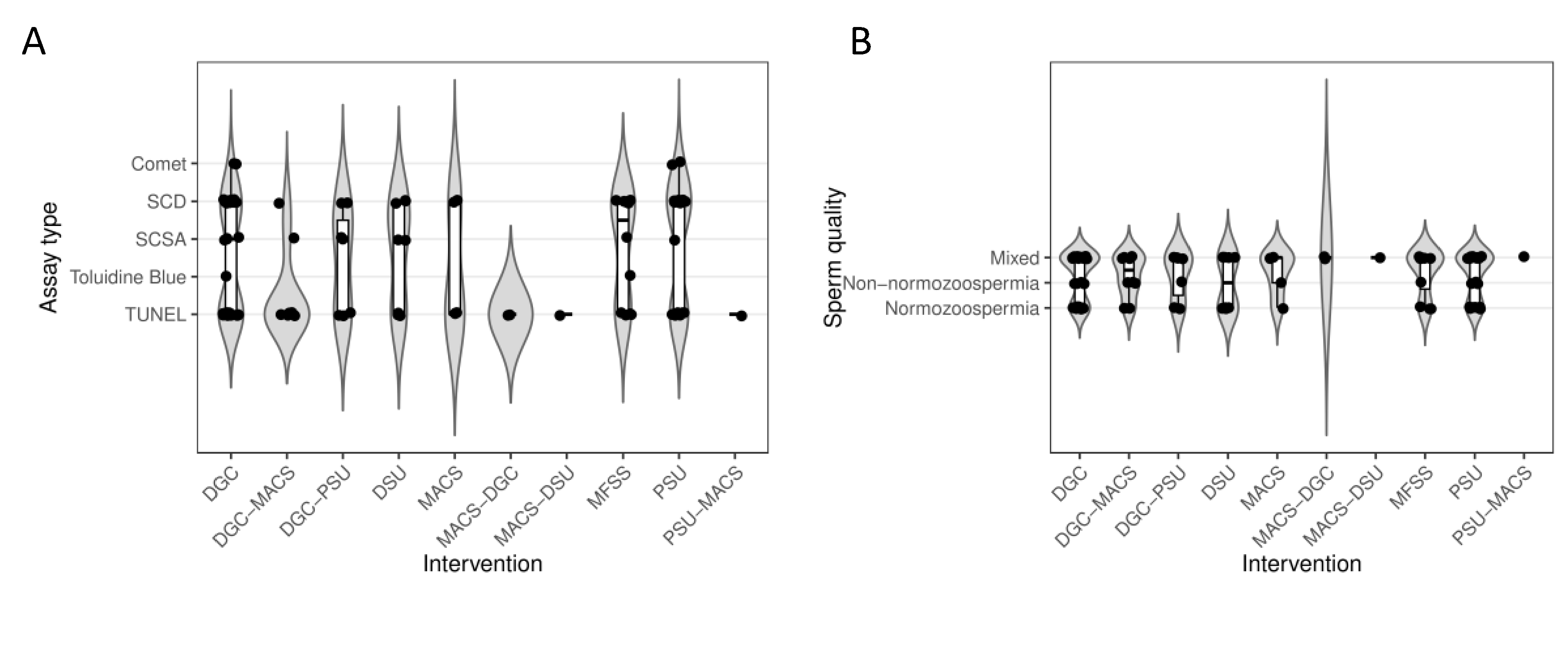

Supplement: Supplementary file 5 [file Image5.tiff]

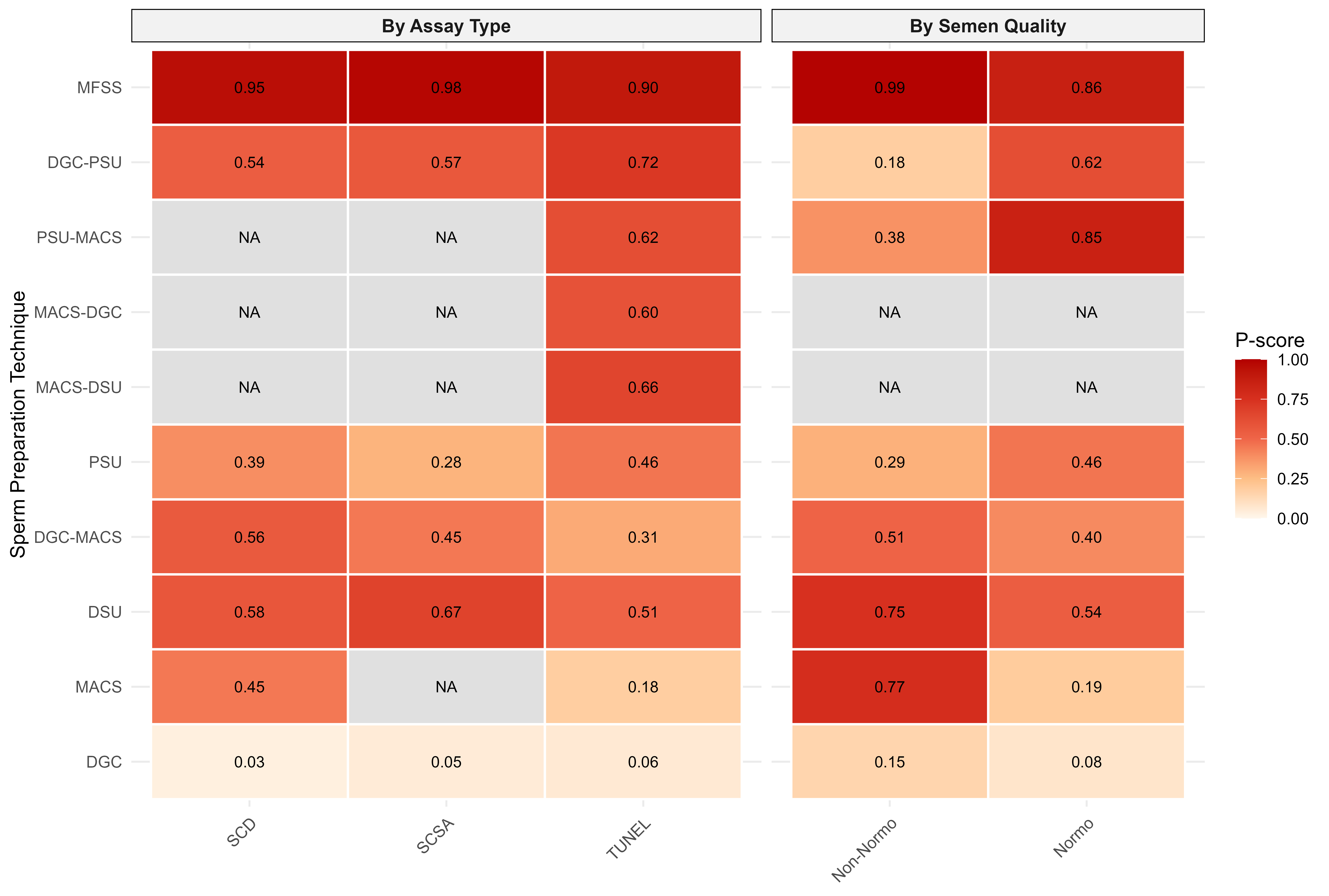

Supplement: Supplementary file 6 [file Image6.tiff]
