# Supplementary material for: Comparative analysis of sperm preparation techniques on DNA fragmentation and clinical outcomes: a network meta-analysis
Source: Front Endocrinol (Lausanne). 2026 Jul 13;17:1817587. doi: 10.3389/fendo.2026.1817587 (PMC13402121; doi:10.3389/fendo.2026.1817587)
Supplement: Supplementary file 7 [file Table1.docx]

**Supplementary Table S1**. Search strategy for network meta-analysis on comparison the effectiveness of sperm preparation technique in reducing sperm DNA fragmentation and improving clinical outcomes.

| Outcomes | Electronic Database | Medical subject headings and keywords |
| --- | --- | --- |
| Sperm DNA fragmentation | PubMed | (Sperm[MeSH] OR Spermatozoa[MeSH] OR sperm*[tiab] OR semen[tiab] ) AND ( DNA Fragmentation[MeSH] OR "sperm DNA fragmentation"[tiab] OR DFI[tiab] ) AND ( DGC[tiab] OR "density gradient"[tiab] OR "density-gradient"[tiab] OR "swim up"[tiab] OR swim-up[tiab] OR SU[tiab] OR MACS[tiab] OR "magnetic activated"[tiab] OR "magnetic sorting"[tiab] OR MFSS[tiab] OR microfluidic*[tiab] OR Zymot[tiab] OR Felix[tiab] ) |
|  | CENTRAL | (sperm* OR semen)AND("DNA fragmentation" OR "sperm DNA fragmentation" OR DFI OR "DNA integrity" OR "DNA damage")AND("density gradient" OR DGC OR "swim up" OR "swim-up" OR SU OR MACS OR "magnetic activated cell sorting" OR "magnetic sorting"  OR MFSS OR "microfluidic" OR "microfluidic sperm sorting") |
|  | Embase | ('spermatozoon'/exp OR sperm:ti,ab OR semen:ti,ab OR 'semen sample*':ti,ab)  AND('dna fragmentation'/exp OR 'sperm dna fragmentation':ti,ab OR 'dna fragmentation index':ti,ab OR dfi:ti,ab)AND('density gradient centrifugation'/exp OR 'density gradient':ti,ab OR dgc:ti,ab OR 'swim up':ti,ab OR swim-up:ti,ab OR su:ti,ab OR macs:ti,ab OR 'magnetic activated cell sorting':ti,ab OR 'magnetic cell sorting':ti,ab OR microfluidic*:ti,ab OR mfss:ti,ab OR microfluidic-chip*:ti,ab OR microfluidic device*:ti,ab) NOT  ('animal'/exp NOT 'human'/exp) |
| Clinical outcomes | PubMed | ("Sperm Preparation Techniques"[Mesh] OR sperm preparation[Title/Abstract]OR sperm selection[Title/Abstract]OR sperm processing[Title/Abstract]OR density gradient centrifug*[Title/Abstract] OR DGC[Title/Abstract]OR swim up[Title/Abstract]OR swim-up[Title/Abstract]OR SU[Title/Abstract]OR magnetic activated cell sorting[Title/Abstract]  OR MACS[Title/Abstract]OR microfluidic*[Title/Abstract]OR MFSS[Title/Abstract])  AND("Pregnancy Outcome"[Mesh] OR "Live Birth"[Mesh]OR "Fertilization"[Mesh]OR "Embryo Implantation"[Mesh]OR "Abortion, Spontaneous"[Mesh] OR pregnancy [Title/Abstract] OR "live birth"[Title/Abstract]OR fertilization[Title/Abstract]OR implantation [Title/Abstract] OR miscarriage[Title/Abstract]) |
|  | CENTRAL | (sperm OR semen) AND (density gradient centrifug* OR DGC OR swim up OR swim-up OR SU OR magnetic activated cell sorting OR MACS OR microfluidic* OR MFSS) |
|  | Embase | ('density gradient centrifugation'/exp OR 'density gradient centrifugation' OR 'swim up technique'/exp OR 'swim up technique' OR 'magnetic activated cell sorting'/exp OR 'magnetic activated cell sorting' OR 'microfluidic device'/exp OR 'microfluidic device') AND ('pregnancy'/exp OR 'pregnancy' OR 'spontaneous abortion'/exp OR 'spontaneous abortion' OR 'fertilization rate'/exp OR 'fertilization rate' OR 'implantation rate'/exp OR 'implantation rate' OR 'live birth rate'/exp OR 'live birth rate') |
