# Supplementary material for: Comparative analysis of sperm preparation techniques on DNA fragmentation and clinical outcomes: a network meta-analysis
Source: Front Endocrinol (Lausanne). 2026 Jul 13;17:1817587. doi: 10.3389/fendo.2026.1817587 (PMC13402121; doi:10.3389/fendo.2026.1817587)
Supplement: Supplementary file 8 [file Table2.docx]

**Supplementary Table S2**. Risk of bias evaluation for non-randomized studies involved in sperm DNA fragmentation using Newcastle -Ottawa Scale.

| Study | Selection^a^ (0-4) | | | | Comparability^b^ (0-2) | Outcome^c^(0-3) | | | Total (0-9) | Rating |
| --- | --- | --- | --- | --- | --- | --- | --- | --- | --- | --- |
|  | Q1 | Q2 | Q3 | Q4 | Q5 | Q6 | Q7 | Q8 |  |  |
| Xue et al., 2014 |  |  |  |  |  |  |  |  | 8 stars | Low risk |
| M.Muratori et al., 2019 |  |  |  |  |  |  |  |  | 7 stars | Moderate |
| Amano et al., 2024 |  |  |  |  |  |  |  |  | 8 stars | Low risk |
| Kim et al., 2017 |  |  |  |  |  |  |  |  | 8 stars | Low risk |
| Amiri et al., 2012 |  |  |  |  |  |  |  |  | 8 stars | Low risk |
| Viswambharan et al., 2020 |  |  |  |  |  |  |  |  | 8 stars | Low risk |
| Jayaraman et  al., 2012 |  |  |  |  |  |  |  |  | 8 stars | Low risk |
| Zhang et al., 2011 |  |  |  |  |  |  |  |  | 8 stars | Low risk |
| Zini et al., 2000 |  |  |  |  |  |  |  |  | 8 stars | Low risk |
| Ahmad et al., 2009 |  |  |  |  |  |  |  |  | 8 stars | Low risk |
| Hardiyanto et al., 2019 |  |  |  |  |  |  |  |  | 8 stars | Low risk |
| Sheibak et al., 2024 |  |  |  |  |  |  |  |  | 8 stars | Low risk |
| Ghaleno et al., 2014 |  |  |  |  |  |  |  |  | 8 stars | Low risk |
| Enciso et al., 2011 |  |  |  |  |  |  |  |  | 8 stars | Low risk |
| Lachaud et al., 2004 |  |  |  |  |  |  |  |  | 8 stars | Low risk |
| Matsuura et al., 2010 |  |  |  |  |  |  |  |  | 8 stars | Low risk |
| Jamil et al., 2023 |  |  |  |  |  |  |  |  | 8 stars | Low risk |
| Volpes et al., 2016 |  |  |  |  |  |  |  |  | 8 stars | Low risk |
| Jackson et al., 2010 |  |  |  |  |  |  |  |  | 8 stars | Low risk |
| T.Degheidy et al., 2014 |  |  |  |  |  |  |  |  | 8 stars | Low risk |
| Lee et al., 2009 |  |  |  |  |  |  |  |  | 8 stars | Low risk |
| H.Zhang et al., 2017 |  |  |  |  |  |  |  |  | 8 stars | Low risk |
| Cakar et al., 2016 |  |  |  |  |  |  |  |  | 8 stars | Low risk |
| Bucar et al., 2014 |  |  |  |  |  |  |  |  | 8 stars | Low risk |
| Nadalini et al., 2014 |  |  |  |  |  |  |  |  | 8 stars | Low risk |
| Bibi et al., 2023 |  |  |  |  |  |  |  |  | 7 stars | Low risk |
| Vahidi et al., 2025 |  |  |  |  |  |  |  |  | 8 stars | Low risk |
| Mateizel et al., 2024 |  |  |  |  |  |  |  |  | 8 stars | Low risk |
| Chi et al., 2016 |  |  |  |  |  |  |  |  | 8 stars | Low risk |
| M Tavalaee et al., 2011 |  |  |  |  |  |  |  |  | 8 stars | Low risk |
| T.S.Berteli et al., 2017 |  |  |  |  |  |  |  |  | 8 stars | Low risk |
| Hsu et al., 2023 |  |  |  |  |  |  |  |  | 8 stars | Low risk |
| Kishi et al., 2015 |  |  |  |  |  |  |  |  | 8 stars | Low risk |
| Mirsanei et al., 2022 |  |  |  |  |  |  |  |  | 8 stars | Low risk |
| Jeseta et al., 2025 |  |  |  |  |  |  |  |  | 8 stars | Low risk |
| Parrella et al., 2019 |  |  |  |  |  |  |  |  | 8 stars | Low risk |
| Traini et al., 2025 |  |  |  |  |  |  |  |  | 8 stars | Low risk |
| Yildiz et al., 2019 |  |  |  |  |  |  |  |  | 7 stars | Moderate |
| Meitei et al., 2021 |  |  |  |  |  |  |  |  | 8 stars | Low risk |
| Le te al., 2022 |  |  |  |  |  |  |  |  | 8 stars | Low risk |

^a^ Selection domain (Q1-Q4): This domain evaluates 1) whether the semen samples included in the study populations were representative of the target clinical population; 2) whether comparison groups were selected appropriately (i.e., different preparation methods performed on the same semen sample); 3) whether the laboratory method for sperm preparation and outcome measurement was adequately described; and 4) whether outcomes were measured on the processed sample.

^b^ Comparability domain Q5: This domain assesses if studies controlled for the typical confounders that may influence sperm quality (such as age, abstinence time, baseline semen quality, and lifestyle factors that may influence DNA fragmentation)

^c^ Outcome domain Q6: This domain assesses whether sperm DNA fragmentation and other sperm parameters were measured using validated laboratory methods (e.g., SCSA, TUNEL, SCD). Q7: This domain assesses whether the follow-up was long enough for outcomes to occur (for DFI outcomes, measurement was considered immediate and therefore adequate). Q

8: This domain assesses the adequacy of follow-up of cohorts (based on the completeness of outcome data and the absence of missing samples).
