# Supplementary material for: Comparative analysis of sperm preparation techniques on DNA fragmentation and clinical outcomes: a network meta-analysis
Source: Front Endocrinol (Lausanne). 2026 Jul 13;17:1817587. doi: 10.3389/fendo.2026.1817587 (PMC13402121; doi:10.3389/fendo.2026.1817587)
Supplement: Supplementary file 9 [file Table3.docx]

**Supplementary Table S3**. CINeMA assessment of certainty of evidence for all network comparisons evaluating the effect of sperm preparation techniques on sperm DNA fragmentation

| Comparison | Number of studies | Within-study bias | Reporting bias | Indirectness | Imprecision | Heterogeneity | Incoherence | Confidence rating |
| --- | --- | --- | --- | --- | --- | --- | --- | --- |
| DGC vs DGC-MACS | 10 | No concerns | Some concerns | No concerns | No concerns | Major concerns | Some concerns | Low |
| DGC vs DGC-PSU | 6 | No concerns | Some concerns | No concerns | No concerns | Major concerns | No concerns | Low |
| DGC vs DSU | 6 | No concerns | Some concerns | No concerns | No concerns | Major concerns | No concerns | Low |
| DGC vs MACS | 4 | No concerns | Some concerns | No concerns | Some concerns | Some concerns | Some concerns | Low |
| DGC vs MACS-DGC | 2 | No concerns | Some concerns | No concerns | No concerns | Major concerns | No concerns | Low |
| DGC vs MFSS | 7 | No concerns | Some concerns | No concerns | No concerns | No concerns | No concerns | Moderate |
| DGC vs PSU | 21 | No concerns | Some concerns | No concerns | No concerns | Major concerns | No concerns | Low |
| DGC vs PSU-MACS | 1 | No concerns | Some concerns | No concerns | Some concerns | Some concerns | No concerns | Low |
| DGC-MACS vs DGC-PSU | 2 | No concerns | Some concerns | No concerns | Some concerns | Some concerns | No concerns | Low |
| DGC-MACS vs MACS | 3 | No concerns | Some concerns | No concerns | Major concerns | No concerns | No concerns | Low |
| DGC-MACS vs MACS-DGC | 2 | No concerns | Some concerns | No concerns | Some concerns | Some concerns | No concerns | Low |
| DGC-MACS vs PSU | 2 | No concerns | Some concerns | No concerns | Major concerns | No concerns | No concerns | Low |
| DGC-MACS vs PSU-MACS | 1 | No concerns | Some concerns | No concerns | Major concerns | No concerns | No concerns | Very low |
| DGC-PSU vs DSU | 2 | No concerns | Some concerns | No concerns | Major concerns | No concerns | Some concerns | Low |
| DGC-PSU vs MACS-DSU | 1 | No concerns | Some concerns | No concerns | Major concerns | No concerns | Major concerns | Very low |
| DGC-PSU vs PSU | 3 | No concerns | Some concerns | No concerns | Some concerns | Some concerns | No concerns | Low |
| DSU vs MFSS | 3 | No concerns | Some concerns | No concerns | No concerns | Major concerns | No concerns | Low |
| DSU vs PSU | 3 | No concerns | Some concerns | No concerns | Some concerns | Some concerns | No concerns | Low |
| MACS vs MACS-DGC | 2 | No concerns | Some concerns | No concerns | Some concerns | Some concerns | No concerns | Low |
| MACS vs MFSS | 1 | No concerns | Some concerns | No concerns | No concerns | Some concerns | No concerns | Low |
| MACS vs PSU | 2 | No concerns | Some concerns | No concerns | Major concerns | No concerns | Major concerns | Very low |
| MFSS vs PSU | 5 | No concerns | Some concerns | No concerns | No concerns | Some concerns | No concerns | Low |
| PSU vs PSU-MACS | 1 | No concerns | Some concerns | No concerns | Major concerns | No concerns | No concerns | Very low |
| DGC vs MACS-DSU | 0 | No concerns | Some concerns | No concerns | Major concerns | No concerns | Major concerns | Very low |
| DGC-MACS vs DSU | 0 | No concerns | Some concerns | No concerns | Some concerns | Some concerns | Major concerns | Very low |
| DGC-MACS vs MACS-DSU | 0 | No concerns | Some concerns | No concerns | Major concerns | No concerns | Major concerns | Very low |
| DGC-MACS vs MFSS | 0 | No concerns | Some concerns | No concerns | No concerns | Major concerns | Major concerns | Very low |
| DGC-PSU vs MACS | 0 | No concerns | Some concerns | No concerns | Some concerns | Some concerns | Major concerns | Very low |
| DGC-PSU vs MACS-DGC | 0 | No concerns | Some concerns | No concerns | Major concerns | No concerns | Major concerns | Very low |
| DGC-PSU vs MFSS | 0 | No concerns | Some concerns | No concerns | Some concerns | Some concerns | Major concerns | Very low |
| DGC-PSU vs PSU-MACS | 0 | No concerns | Some concerns | No concerns | Major concerns | No concerns | Major concerns | Very low |
| DSU vs MACS | 0 | No concerns | Some concerns | No concerns | Some concerns | Some concerns | Major concerns | Very low |
| DSU vs MACS-DGC | 0 | No concerns | Some concerns | No concerns | Major concerns | No concerns | Major concerns | Very low |
| DSU vs MACS-DSU | 0 | No concerns | Some concerns | No concerns | Major concerns | No concerns | Major concerns | Very low |
| DSU vs PSU-MACS | 0 | No concerns | Some concerns | No concerns | Major concerns | No concerns | Major concerns | Very low |
| MACS vs MACS-DSU | 0 | No concerns | Some concerns | No concerns | Major concerns | No concerns | Major concerns | Very low |
| MACS vs PSU-MACS | 0 | No concerns | Some concerns | No concerns | Major concerns | No concerns | Major concerns | Very low |
| MACS-DGC vs MACS-DSU | 0 | No concerns | Some concerns | No concerns | Major concerns | No concerns | Major concerns | Very low |
| MACS-DGC vs MFSS | 0 | No concerns | Some concerns | No concerns | Some concerns | Some concerns | Major concerns | Very low |
| MACS-DGC vs PSU | 0 | No concerns | Some concerns | No concerns | Major concerns | No concerns | Major concerns | Very low |
| MACS-DGC vs PSU-MACS | 0 | No concerns | Some concerns | No concerns | Major concerns | No concerns | Major concerns | Very low |
| MACS-DSU vs MFSS | 0 | No concerns | Some concerns | No concerns | Major concerns | No concerns | Major concerns | Very low |
| MACS-DSU vs PSU | 0 | No concerns | Some concerns | No concerns | Major concerns | No concerns | Major concerns | Very low |
| MACS-DSU vs PSU-MACS | 0 | No concerns | Some concerns | No concerns | Major concerns | No concerns | Major concerns | Very low |
| MFSS vs PSU-MACS | 0 | No concerns | Some concerns | No concerns | Major concerns | No concerns | Major concerns | Very low |

CINeMA framework was used to evaluate confidence in the 45 network comparisons (25 direct and 20 indirect). A value of “0” in the “number of studies” column indicates an indirect comparison. In the reporting bias domain, all comparisons were judged as having some concerns, as most included studies lacked preregistration and the comparison-adjusted funnel plot showed significant small study-effects (Egger test p<0.001). Imprecision domain showed major concerns across several comparisons, particularly among the indirect comparisons. This is because indirect estimates rely on multi-step evidence chains that accumulate statistical uncertainty, resulting in wide confidence intervals relative to the prespecified clinically difference (DFI MD=3%). Major concerns were also observed in the incoherence domain. This is in line with the global evidence Q statistic test. These limitations had minimal influence on the key treatment contrasts, which was predominantly supported by direct evidence. The confidence ratings were determined by downgrading from high certainty based on the number of severity of concerns across CINeMA domains.

**Abbreviations**: DGC=density gradient centrifugation; PSU=Pellet Swim-Up; DSU= Direct Swim-Up; DGC-PSU= Swim-Up after DGC (treated as PSU in analysis); MACS=magnetic-activated cell sorting; MACS-DGC, DGC-MACS, PSU-MACS, MACS-WSU=sequential methods; MFSS= Microfluidic sperm sorting.
