# Supplementary material for: Comparative analysis of sperm preparation techniques on DNA fragmentation and clinical outcomes: a network meta-analysis
Source: Front Endocrinol (Lausanne). 2026 Jul 13;17:1817587. doi: 10.3389/fendo.2026.1817587 (PMC13402121; doi:10.3389/fendo.2026.1817587)
Supplement: Supplementary file 10 [file Table4.docx]

| Design-based Q statistic, comparisons and overall statement | Q statistic | Degree of freedom | *p*-value |
| --- | --- | --- | --- |
| Design-specific decomposition of within-design Q statistic | | | |
| DGC vs PSU | 1306.99 | 13 | <0.0001 |
| DGC vs MFSS | 37.52 | 4 | <0.0001 |
| DGC vs DGC-MACS | 8.10 | 3 | 0.0441 |
| MFSS vs PSU | 3.80 | 1 | 0.0512 |
| DGC vs DSU | 0.03 | 1 | 0.8622 |
| DGC vs DGC-MACS vs MACS vs MACS-DGC | 0.12 | 3 | 0.9895 |
| Between-design Q statistic after detaching of single designs | | | |
| MFSS vs PSU | 933.28 | 30 | <0.0001 |
| DGC vs MFSS | 1209.76 | 30 | <0.0001 |
| DGC vs PSU | 1452.59 | 30 | <0.0001 |
| DGC vs DGC-MACS | 2870.89 | 30 | 0 |
| DGC vs DGC-PSU | 2851.74 | 30 | 0 |
| DGC vs MFSS | 2751.27 | 30 | 0 |
| DGC vs DGC-MACS vs DGC-PSU | 2871.92 | 30 | 0 |
| DGC vs DGC-MACS vs DGC-PSU vs PSU | 2870.69 | 30 | 0 |
| DGC vs DGC-MACS vs MACS | 2723.25 | 30 | 0 |
| DGC vs DGC-PSU vs DSU vs PSU | 2797.14 | 30 | 0 |
| DGC vs DGC-PSU vs PSU | 2865.79 | 30 | 0 |
| DGC vs DSU vs MFSS | 2516.77 | 30 | 0 |
| DGC vs DSU vs PSU | 2726.93 | 30 | 0 |
| DGC vs MACS vs PSU | 2849.64 | 30 | 0 |
| DGC vs MFSS vs PSU | 2772.73 | 30 | 0 |
| DSU vs MFSS vs PSU | 2799.07 | 30 | 0 |
| MACS vs MFSS vs PSU | 2825.89 | 30 | 0 |

**Supplementary Table S4**. The global inconsistency test for network meta-analysis on sperm

DNA fragmentation.

The global inconsistency test was used Q statistic. The within design Q test showed significant heterogeneity in direct comparisons between DGC vs PSU (*P<0.001*), DGC vs MFSS (*p <0.0001*) and DGC vs DGC-MACS (*P=0.04*)), which counted for most variability among direct contrasts. The between design Q statistic remained significant after sequential detachment of each design, indicating that the network continued to exhibit inconsistency even after removal of any individual design. (Design with *p < 0.05* considered statistically significant).

**Abbreviations**: DGC=density gradient centrifugation; PSU=Pellet Swim-Up; DSU= Direct Swim-Up; DGC-PSU= Swim-Up after DGC (treated as PSU in analysis); MACS=magnetic-activated cell sorting; MACS-DGC, DGC-MACS, PSU-MACS, MACS-WSU=sequential methods; MFSS= Microfluidic sperm sorting.
