# Supplementary material for: Comparative analysis of sperm preparation techniques on DNA fragmentation and clinical outcomes: a network meta-analysis
Source: Front Endocrinol (Lausanne). 2026 Jul 13;17:1817587. doi: 10.3389/fendo.2026.1817587 (PMC13402121; doi:10.3389/fendo.2026.1817587)
Supplement: Supplementary file 11 [file Table5.docx]

Supplementary Table S5. Node-splitting inconsistency assessment for network meta-analysis on sperm DNA fragmentation.

| **Comparisons** | **Direct estimates** | **Indirect estimates** | **Network estimate** | **P-value** |
| --- | --- | --- | --- | --- |
| DGC-MACS vs DGC | -2.48 | -17.00 | -4.02 | 0.003* |
| DGC-PSU vs DGC | -6.92 | -8.98 | -7.37 | 0.64 |
| DSU vs DGC | -7.73 | -3.55 | -6.33 | 0.25 |
| MACS vs DGC | -0.05 | -7.71 | -2.77 | 0.08 |
| MACS-DGC vs DGC | -6.23 | -10.39 | -7.41 | 0.55 |
| MFSS vs DGC | -13.36 | -9.55 | -11.71 | 0.21 |
| PSU vs DGC | -4.09 | -4.04 | -4.08 | 0.98 |
| PSU-MACS vs DGC | -4.80 | -13.05 | -7.35 | 0.42 |
| DGC-MACS vs DGC-PSU | -0.80 | 6.17 | 3.35 | 0.12 |
| DGC-MACS vs MACS | -3.94 | 2.26 | -1.24 | 0.18 |
| DGC-MACS vs MACS-DGC | 2.59 | 5.76 | 3.39 | 0.66 |
| DGC-MACS vs PSU | -2.74 | 0.77 | 0.06 | 0.42 |
| DGC-MACS vs PSU-MACS | 3.20 | 3.64 | 3.33 | 0.96 |
| DGC-PSU vs DSU | -6.00 | 2.67 | -1.03 | 0.06 |
| DGC-PSU vs PSU | -1.02 | -5.36 | -3.28 | 0.27 |
| DSU vs MFSS | 4.53 | 6.09 | 5.37 | 0.69 |
| DSU vs PSU | -0.50 | -3.34 | -2.25 | 0.45 |
| MACS vs MACS-DGC | 6.91 | -2.05 | 4.63 | 0.24 |
| MACS vs MFSS | 5.69 | 9.86 | 8.93 | 0.48 |
| MACS vs PSU | -5.69 | 5.62 | 1.31 | 0.01* |
| MFSS vs PSU | -7.61 | -7.63 | -7.62 | 0.99 |
| PSU vs PSU-MACS | 6.85 | -2.08 | 3.27 | 0.37 |

Node-splitting analyses were performed to evaluate local inconsistency by comparing direct versus indirect evidence within each closed loop. Inconsistency was detected primarily in the comparisons of DGC-MACS vs DGC (*p=0.0037*) and MACS vs PSU (*p= 0.01*), indicating disagreement between direct and indirect estimates for these pairs. All other comparisons showed no statistically significant inconsistency.

* *p < 0.05* considered statistically significant

Abbreviations: DFI=DNA fragmentation index; NA= not applicable. DGC=density gradient centrifugation; PSU=Pellet Swim-Up; DSU= Direct Swim-Up; DGC-PSU= Swim-Up after DGC (treated as PSU in analysis); MACS=magnetic-activated cell sorting; MACS-DGC, DGC-MACS, PSU-MACS, MACS-WSU=sequential methods; MFSS= Microfluidic sperm sorting
