# Supplementary material for: Comparative analysis of sperm preparation techniques on DNA fragmentation and clinical outcomes: a network meta-analysis
Source: Front Endocrinol (Lausanne). 2026 Jul 13;17:1817587. doi: 10.3389/fendo.2026.1817587 (PMC13402121; doi:10.3389/fendo.2026.1817587)
Supplement: Supplementary file 12 [file Table6.docx]

**Supplementary Table S6**. Assessment of transitivity: distribution of participant characteristics and sperm DNA fragmentation measurement methods across sperm preparation techniques

| Treatment | No. studies | Normozoospemia | Non-normozoospermia | mixed | SCSA | TUNEL | SCD | Other |
| --- | --- | --- | --- | --- | --- | --- | --- | --- |
| DGC | 37 | 16 (43.2%) | 7 (18.9%) | 14 (37.8%) | 3 (8.1%) | 17 (45.9%) | 14 (37.8%) |  |
| MACS | 5 | 1 (20.0%) | 1 (20.0%) | 2 (40.0%) | 0 (0%) | 3 (60.0%) | 2 (40.0%) |  |
| MFSS | 9 | 4 (33.3%) | 3 (33.3%) | 3 (33.3%) | 0 | 2 (22.2%) | 6 (66.7%) | 1 (11.1%) |
| PSU | 24 | 13 (50.0%) | 6 (25.0%) | 8 (33.3%) | 1 (4.2%) | 9 (37.5%) | 12 (50.0%) |  |
| DSU | 7 | 3 (28.6%) | 1 (14.3%) | 3 (42.9%) | 1 (14.3%) | 3 (42.9%) | 3 (42.9%) |  |
| DGC-MACS | 10 | 3 (30.0%) | 3 (30.0%) | 3 (30.0%) | 1 (10.0%) | 8 (80.0%) | 1 (10.0%) |  |
| MACS-DGC | 2 | 0 | 0 | 1 (50.0%) | 0 | 2 (100%) | 0 |  |
| DGC-PSU | 7 | 3 (42.9%) | 2 (28.6%) | 4 (57.1%) | 2 (28.6%) | 3 (42.9%) | 2 (28.6%) |  |
| MACS-DSU | 1 | 0 | 0 | 1 (100%) | 0 | 1 (100%) | 0 |  |
| PSU-MACS | 1 | 1 (100%) | 1 (100%) | 0 | 0 | 1 (100%) | 0 |  |

**Abbreviations**: DGC=density gradient centrifugation; PSU=Pellet Swim-Up; DSU= Direct Swim-Up; DGC-PSU= Swim-Up after DGC (treated as PSU in analysis); MACS=magnetic-activated cell sorting; MACS-DGC, DGC-MACS, PSU-MACS, MACS-WSU=sequential methods; MFSS= Microfluidic sperm sorting. TUNEL= Terminal deoxynucleotidyl transferase dUTP nick end labeling; SCD=sperm chromatin dispersion; SCSA=sperm chromatin structure assay;
