# Supplementary material for: Comparative analysis of sperm preparation techniques on DNA fragmentation and clinical outcomes: a network meta-analysis
Source: Front Endocrinol (Lausanne). 2026 Jul 13;17:1817587. doi: 10.3389/fendo.2026.1817587 (PMC13402121; doi:10.3389/fendo.2026.1817587)
Supplement: Supplementary file 13 [file Table7.docx]

**Supplementary Table S7**. Characteristics of female age and ovarian reserve in included RCTs for clinical outcome analysis

| Study | Comparisons | Female age | | Female age difference | AMH reported |
| --- | --- | --- | --- | --- | --- |
|  |  | Control | Intervention |  |  |
| Dodson et al.,1998 | NA | | | | |
| Carrell et al., 1998 | NA | | | | |
| Troya et al., 2015 | DGC VS MACS | 37.07±0.52 | 36.94±0.81 | NS | Not reported |
| Ozaltin et al.,2023 | DGC VS MFSS | 35.2±6.2 | 34.9±5.0 | NS | Not reported |
| Ravina et al.,2022 | NA | | | | |
| Romany et al.,2014 | SU VS MACS | 25.4±3.7 | 25.7±4.5 | NS | Not reported |
| Mei et al., 2022 | DGC-SU VS MACS | 30.4±3.7 | 30.1 ± 4.5 | NS | Not reported |
| Anbari et al., 2021 | SU VS MFSS | 30.76 ±3.99 | 32.4 ± 6.02 | NS | Not reported |
| Hozyen et al., 2021 | DGC VS MACS | 29.7 ± 4.5 | 29.9 ± 4.2 | NS | Reported |
| Godiwala et al.,2023 | NA | | | | |
| Yetkinel et al.,2018 | SU VS MFSS | 28.21±3.31 | 28.61±2.96 | NS | Not reported |
| Duong et al.,2024 | DGC VS SU | 31.3±3.8 | 31.2 ±4.2 | NS | Not reported |
| Yildiz et al., 2019 | DGC VS MFSS | 31.49±0.5 | 31.07±0.3 | NS | Not reported |
| Ziarati et al.,2017 | DGC VS MACS | 31.6±3.84 | 30.21±4.08 | NS | Not reported |

Abbreviations: NA= not applicable; NS= no statistically significantly difference; DGC=density gradient centrifugation; SU= Swim-Up; DGC-SU= combination of DGC with SU; MACS=magnetic-activated cell sorting; MFSS= Microfluidic sperm sorting
