# Supplementary material for: Comparative analysis of sperm preparation techniques on DNA fragmentation and clinical outcomes: a network meta-analysis
Source: Front Endocrinol (Lausanne). 2026 Jul 13;17:1817587. doi: 10.3389/fendo.2026.1817587 (PMC13402121; doi:10.3389/fendo.2026.1817587)
Supplement: Supplementary file 14 [file DataSheet1.docx]

**Supplementary Figure S1.** PRISMA flow diagram of article screening for clinical outcomes.

Abbreviations: CENTRAL= Cochrane Central Register of Controlled Trials

**Supplementary Figure S2**: Risk of Bias evaluation for studies included in clinical outcomes analyses. D1: Randomization process; D2: Deviations from the intended interventions; D3: Missing outcome data; D4: Measurement of the outcome; D5: Selection of the reported result;

Low risk some concerns High risk

**Supplementary Figure S3.** Forest plots of pairwise meta-analysis comparing the effectiveness of sperm preparation techniques in reducing sperm DNA fragmentation.

Random-effect pairwise meta-analyses were performed for nine comparisons. The sperm preparation techniques involved in each comparison are indicated at the top of each forest plot. In forest plots e—i, density gradient centrifugation (DGC) was used as the reference, and the comparators demonstrated significant lower DNA fragmentation index (DFI). In forest plots a, c and d, pellet swim-up (PSU) was used as the reference; lower DFI was observed with the comparators, although statistically significant was reached only for microfluidic sperm sorting (MFSS). In forest plot b, direct swim-up (DSU) was used as the reference, and MFSS showed a significant reduction in DFI. Negative standardized mean difference (MD) indicates a reduction in sperm DNA fragmentation favoring the comparator.

Abbreviations: DGC-PSU= Swim-Up after DGC (treated as PSU in analysis); PSU= Pellet swim-up; MACS=magnetic-activated cell sorting; DGC-MACS= MACS after DGC; PSU-MACS= MACS after PSU; CI=confidential interval.

**Supplementary Figure S4.** Comparation-adjusted funnel plot for the network meta-analysis comparing the effectiveness of sperm preparation techniques in reducing sperm DNA fragmentation.

Each dot represents a direct comparison from the included studies, centered relative to its comparison-specific mean effect. The vertical line marked the pooled effect estimate, and the dashed triangular region shows the expected 95% confidence limits. The Egger’s regression test evaluated funnel plot asymmetry and the result (Supplementary Figure S4) didn’t show statistically significant asymmetry (*p*=0.33), suggesting no clear evidence of publication bias.

Abbreviations: DGC=density gradient centrifugation; PSU=Pellet Swim-Up; DSU= Direct Swim-Up; DGC-PSU= Swim-Up after DGC (treated as PSU in analysis); MACS=magnetic-activated cell sorting; DGC-MACS= MACS after DGC; PSU-MACS= MACS after PSU; MFSS=Microfluidic sperm sorting; CI=confidential interval.

**Supplementary Figure S5:** Transitivity assessment comparing the distribution of sperm quality and assay type across interventions in the network meta-analysis of sperm DNA fragmentation.

(A) violin and box plot compared the distribution of assay types used for DNA fragmentation index (DFI) measurement across all interventions. The results indicated that the distribution of assay type for DFI measurement were comparable.

(B) violin and box plot showed the distribution of sperm quality—normozoospermia, non-normozoospermia and mixed—were comparable across the interventions. *Mixed* indicates studies that include both normozoospermic and non-normozoospermic samples without reporting sperate outcomes for each semen-quality category.

Abbreviations: DGC=density gradient centrifugation; PSU=Pellet Swim-Up; DSU= Direct Swim-Up; DGC-PSU= Swim-Up after DGC (treated as PSU in analysis); MACS=magnetic-activated cell sorting; DGC-MACS= MACS after DGC; PSU-MACS= MACS after PSU; MFSS=Microfluidic sperm sorting;

**Supplementary Figure S6.** SUCRA(P-score) analyses for the subgroup network meta-analyses.

The heatmap presents the SUCRA (P-score) rankings for the comparative effectiveness of sperm preparation techniques in reducing DNA fragmentation, stratified by DNA fragmentation assay type (TUNEL, SCSA and SCD) and semen quality (Normozoospermic and Non-normozoospermic). Higher P-score values reflect a greater likelihood that a given technique is among the most effective in reducing DNA fragmentation within each subgroup. Cells labeled NA indicate treatment nodes that were not included in specific subgroup networks due to a lack of available studies.

Abbreviations: DGC=density gradient centrifugation; PSU=Pellet Swim-Up; DSU= Direct Swim-Up; DGC-PSU= Swim-Up after DGC (treated as PSU in analysis); MACS=magnetic-activated cell sorting; DGC-MACS= MACS after DGC; PSU-MACS= MACS after PSU; MFSS=Microfluidic sperm sorting; NA=not applicable.
